# Modulation of the peripheral blood transcriptome by the ingestion of probiotic yoghurt and acidified milk in healthy, young men

**DOI:** 10.1371/journal.pone.0192947

**Published:** 2018-02-28

**Authors:** Kathryn J. Burton, Grégory Pimentel, Nadine Zangger, Nathalie Vionnet, Jocelyne Drai, Philip G. McTernan, François P. Pralong, Mauro Delorenzi, Guy Vergères

**Affiliations:** 1 Service of Endocrinology, Diabetes and Metabolism, Lausanne University Hospital, Lausanne, Switzerland; 2 Federal Department of Economic Affairs, Education and Research EAER, Agroscope, Berne, Switzerland; 3 SIB Swiss Institute of Bioinformatics, Lausanne, Switzerland; 4 Centre Hospitalier Lyon-Sud, Laboratoire de Biochimie, Pierre-Bénite, France; 5 Equipe Inserm CarMeN U1060, Faculté de Médecine LYON SUD – BP 12, Pierre Bénite, France; 6 School of Science and Technology, Nottingham Trent University, Nottingham, United Kingdom; University of São Paulo, BRAZIL

## Abstract

The metabolic health benefits of fermented milks have already been investigated using clinical biomarkers but the development of transcriptomic analytics in blood offers an alternative approach that may help to sensitively characterise such effects. We aimed to assess the effects of probiotic yoghurt intake, compared to non-fermented, acidified milk intake, on clinical biomarkers and gene expression in peripheral blood. To this end, a randomised, crossover study was conducted in fourteen healthy, young men to test the two dairy products. For a subset of seven subjects, RNA sequencing was used to measure gene expression in blood collected during postprandial tests and after two weeks daily intake. We found that the postprandial response in insulin was different for probiotic yoghurt as compared to that of acidified milk. Moreover changes in several clinical biomarkers were associated with changes in the expression of genes representing six metabolic genesets. Assessment of the postprandial effects of each dairy product on gene expression by geneset enrichment analysis revealed significant, similar modulation of inflammatory and glycolytic genes after both probiotic yoghurt and acidified milk intake, although distinct kinetic characteristics of the modulation differentiated the dairy products. The aryl hydrocarbon receptor was a major contributor to the down-regulation of the inflammatory genesets and was also positively associated with changes in circulating insulin at 2h after yoghurt intake (*p* = 0.05). Daily intake of the dairy products showed little effect on the fasting blood transcriptome. Probiotic yoghurt and acidified milk appear to affect similar gene pathways during the postprandial phase but differences in the timing and the extent of this modulation may lead to different physiological consequences. The functional relevance of these differences in gene expression is supported by their associations with circulating biomarkers.

## Introduction

Fermentation is a widely used method for processing dairy milk that results in the transformation of the milk by the action of lactic acid bacteria. The changes that accompany milk fermentation include nutrient, chemical and physical modifications of the milk matrix. Lactic acid bacteria are used for milk fermentation for their capacity to metabolise lactose and galactose to lactic acid [1], but their actions during fermentation extend beyond carbohydrate metabolism with important consequences for the amino acid, mineral and vitamin composition of the milk [2]. In addition, some lactic bacteria affect the microbiota of the consumer as they are probiotics, live microorganisms that when consumed in adequate quantities, provide a health benefit to the host [3]. Thus the many health benefits that have been described for fermented milks may be attributed to the effects of the fermentation-specific changes of the milk on digestion and metabolism, and/or the interactions of the bacterial strains with the gut microbiota [4]. The most established effect of milk fermentation on metabolism is in the specific case of lactose metabolism in populations that do not have the genotype for lactase persistence; the prior hydrolysis of lactose during the fermentation process promotes the digestibility of dairy products [5, 6]. The wider consequences of milk fermentation on host metabolism have also been investigated; in postprandial studies differential effects of fermented compared to non-fermented dairy milks have been observed for glycemia and insulinemia [7] as well as lipidemia [8] and protein flux [9]. Calcium bioavailability has been investigated in dairy products but does not appear to depend upon milk fermentation [10, 11]. The health benefits of fermented dairy intake are not limited to effects on postprandial metabolism; the daily intake of fermented dairy foods (in particular those containing probiotic bacteria) has been associated with lowering lipid parameters [12], regulation of glycemia [13–16] and a reduction in circulating parameters of inflammation [17–21].

Whilst many studies have sought to investigate the impact of diet on individual metabolic pathways, the emerging field of ‘nutrigenomics’ has increasingly been applied to study the interplay between genes, diet, metabolites and dietary consequences in health and disease [22]. Due to the development of high-throughput technologies, whole blood transcriptomics can offer a rapid, relatively non-invasive approach to study the changes in gene expression in blood that take place in response to dietary intake. Indeed, in the recent work of Petrov *et al*, the value of using whole blood to identify transcript-based biomarkers of nutritional status and metabolic health was demonstrated [23]. Transcriptomic analysis, like other ‘-omic’ approaches, facilitates a broader analysis than classical blood biomarkers that focus on a limited selection of surrogate markers of metabolism. Of note, the blood transcriptome has been shown to reflect gene expression in other tissues and thus may capture the wider consequences of diet on the body [24]. Furthermore, in the recent work of Bartel *et al*., the fasting whole blood transcriptome was closely associated with the fasting circulating metabolome and implied metabolic processes [25]. The relevance of nutrient-gene interactions for dairy research has already been identified and explored in human trials [18, 26]. Notably, Sagaya *et al*. compared the acute intake of yoghurt and of a non-fermented, acidified milk on gene expression of blood cells, and reported broadly similar effects for the two dairy products, including the modulation of inflammatory processes [26]. Conversely, a specific role for probiotic bacteria added to yoghurt on gene expression of the inflammatory related gene, RAR-related orphan receptor gamma, was suggested by Zarrati *et al*. [18] in a study that compared standard yoghurt with probiotic yoghurt.

The analysis of transcriptome changes in blood to assess the acute impact of dietary intake can be considered as a type of *in vivo* cellular experiment that is characterised by a change in nutrient composition of the media (serum) in which the cell (blood cells) are active and the response of the cells to this change is measured. In contrast to the classical *in vitro* cellular experiment, the human model reflects the true dynamics of nutrient digestion in the gastrointestinal tract, including the complex interactions with the gut microbiota, transport and processing by the intestinal cells and metabolism by the liver, before the active metabolites act upon the blood cells.

This approach could thus support understanding of how the intake of fermented dairy foods, especially those containing probiotic strains, can affect metabolism and physiological processes that may play a role in the health qualities associated with these foods. In this study, we use whole blood transcriptomics together with classical circulating biomarkers to explore in an *in vivo* human model the postprandial and short-term effects of probiotic yoghurt compared to non-fermented, acidified milk.

## Materials and methods

### Ethics statement

All procedures were completed in accordance with the ethical standards of the responsible committee on human experimentation and with the guidelines laid down in the Helsinki Declaration. Ethical approval for the study was received from the regional committee for human experimentation (approval number: 392/13, Vaud, Switzerland) and written informed consent obtained from all participants. This trial was retrospectively registered at clinicaltrial.gov on July 21, 2014 (registration number: NCT02230345).

### Subjects

Participants in the study were healthy, young men with a mean (±SEM) age of 24.6 ± 4.7 years and mean (±SEM) BMI of 21.8 ± 1.8 kg/m^2^ recruited from the Lausanne region by poster campaign. Exclusion criteria included dietary intolerances, abnormal clinical biochemistry, acute or chronic illness, use of medication, and use of antibiotics in the six months prior to start of the study. One subject was excluded *post hoc*, following analysis of the microbiota samples of the study [21], thus clinical biochemistry, inflammatory parameters and appetite sensations were assessed for the thirteen remaining subjects. Gene expression was assessed in whole blood samples from a subset of seven subjects as this sample size has previously been shown to be adequate to obtain significant and physiologically meaningful postprandial results in a study that used lower caloric doses of dairy products [26].

### Study design

**Fig 1:**
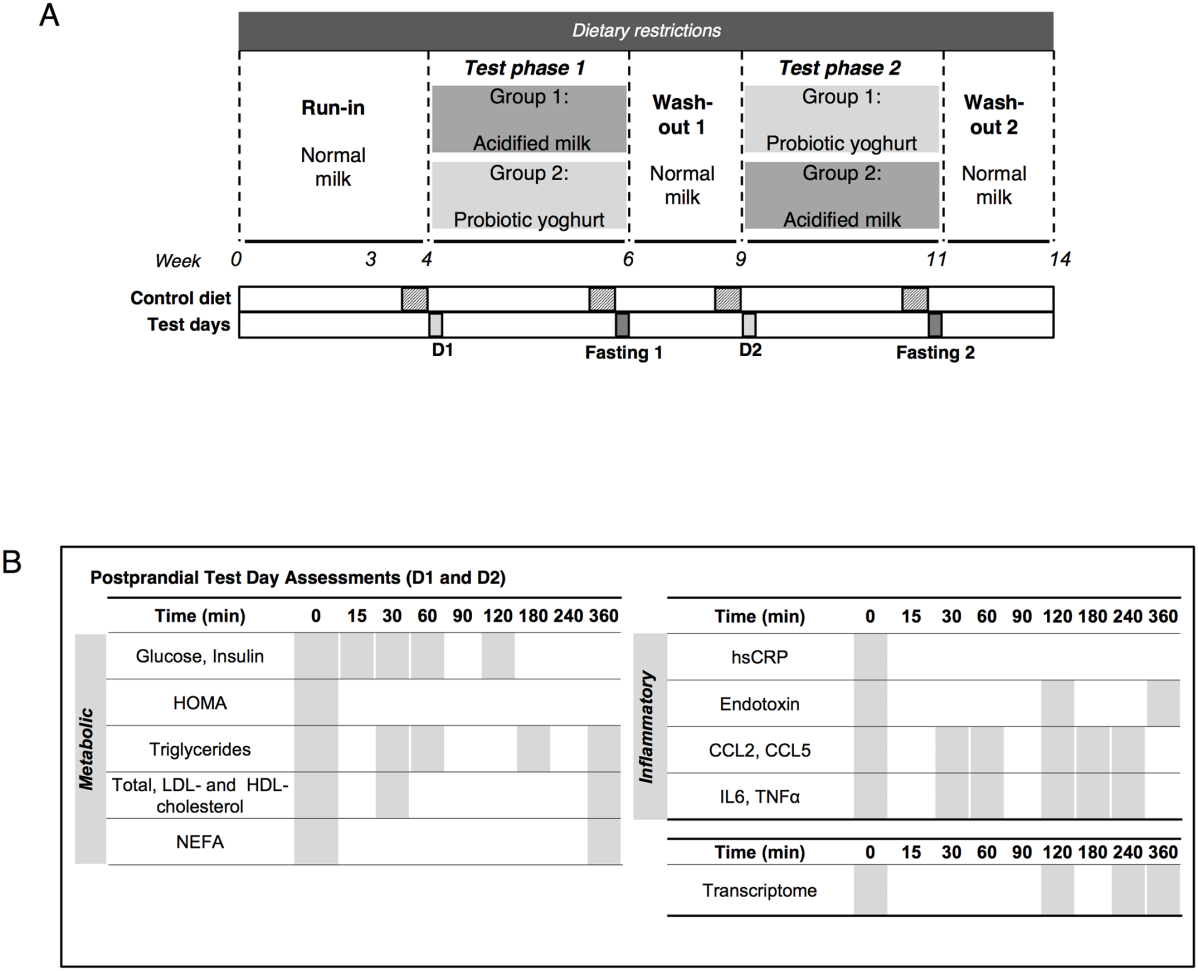
Overview of crossover study design (adapted from Burton *et al*., 2017 [21]). A. Probiotic yoghurt and acidified milk were consumed during two test phases. Postprandial dairy tests (D1 and D2) were completed at the beginning of each test phase and fasting tests were completed after two weeks intake of each product (Fasting 1 and 2). Run-in and wash-out periods respectively preceded and followed the two test phases. Three-day controlled diets were provided prior to all test days and dairy intake was restricted during all study phases. B. Blood sampling on D1 and D2 assessed metabolic, inflammatory and gene expression changes in the six-hour period following dairy intake. All parameters were assessed for the fasting tests. Abbreviations: HOMA, homeostatic model assessment; NEFA, non-esterified fatty acids; hsCRP, high sensitivity C-reactive protein; LPS, lipopolysaccharide; CCL2, chemokine ligand 2; CCL5, chemokine ligand 5; IL6, interleukin 6; TNFα, tumor necrosis factor alpha.

A randomised, double blind, crossover study design was used to evaluate effects of two dairy products, probiotic yoghurt and acidified milk, as described previously [21] (Fig 1A). The effect of a single intake of the assigned dairy product (800 g) was assessed by a postprandial test (D1 and D2). Each postprandial test was completed after an overnight fast and required participants to consume the designated dairy product within a period of 15 min. Venous blood sampling was completed in the fasting state and during the six-hour postprandial period following dairy intake. No intake except *ad libitum* water intake was permitted during the postprandial test. Blood sampling was completed for assessment of clinical biochemistry and inflammatory parameters as well as for transcriptome analysis (Fig 1B, full study design reported in Burton *et al*., 2017 [21]). A visual analogue scale questionnaire (adapted from Flint *et al*., 2000 [27], S1 Table) was completed by the subjects during the postprandial tests (0, 0.5, 1, 1.5, 2, 4 and 6 h) to assess hunger, satiety, prospective food consumption, appetite and subjective state of ease. The questionnaire required a response to six questions that was indicated on a 100 mm single line scale within the Adaptive Visual Analogue Scale program [28]. The postprandial test initiated the short-term administration phase of the study that comprised the bi-daily intake of the assigned dairy product over two weeks (2 x 200 g/d). The effect of this short-term intervention was assessed by fasting sampling (Fasting 1 and 2). Run-in and wash-out phases that included dietary restrictions (as detailed previously [21]) and fixed volumes of normal (non-acidified) milk (400 ml/d), preceded and separated the two exposures. All clinical test days of the study were carried out at the Centre of Clinical Research, University Hospital of Lausanne.

#### Dairy products

All dairy products used in the study were sourced from a single batch of full fat (3.5%) provided by Emmi AG, Luzern. The two test products were a probiotic yoghurt and a milk, acidified by the addition of D-(+)-glucono-delta-lactone (2%) to replicate the physical and chemical characteristics of the yoghurt. The probiotic yoghurt was fermented by *Lactobacillus delbrueckii* spp. *bulgaricus* and *Streptococcus thermophilus* (Thermophilic Yoflex^®^ culture, CHR Hansen, Denmark), and *Lactobacillus rhamnosus* GG (LGG) (ATCC 53103- Culture Collection of the University of Goteborg, Sweden, ref. CCUG 34291), (full nutrient composition and methods have been reported previously [21]).

### Sampling

Serum, plasma and whole blood Paxgene^®^ samples were collected for each postprandial dairy test (fasted and postprandial) and after each chronic exposure (fasted) (Fig 1B). Postprandial whole blood samples were collected at three selected time points per postprandial dairy test (2, 4 and 6 h), while serum and plasma were collected at nine time points postprandially [21]. Metabolic biomarkers (glucose, insulin, triglycerides, cholesterol profile, non-esterified fatty acids) and inflammatory biomarkers (high sensitivity C-reactive protein (hsCRP), lipopolysaccharide (LPS), chemokine ligand 2 (CCL2), chemokine ligand 5 (CCL5), interleukin 6 (IL6), tumor necrosis factor alpha (TNFα)) were analysed in plasma and assayed as described previously [21].

### RNA processing

RNA was extracted from whole blood PAXgene^®^ samples using the “PAXgene^®^ Blood miRNA kit” (Qiagen^®^ EmbH, Germany) as per manufacturer’s protocol. Quantification and quality control assessments were completed using the Nanodrop^™^ 1000 (Thermo Scientific, USA) and the Fragment analyzer^™^ instrument (Advanced Analytical^®^ Technologies). For downstream analyses, samples were required to meet quality criteria of A260/A280 ratio > 1.8 and RQN > 8.0. To remove detected impurities, a standard ethanol purification protocol was applied to all samples: 10 μl 3 M sodium acetate (pH 5.2) (Millipore^™^, USA), 2 μl of 5 mg/ml glycogen (Ambion^®^, Life Technologies, USA) and 300 μl of 99% ethanol were added to each 100 μl samples (dilution of samples with RNAse-free water) prior to vortex and incubation at -80°C for 2 h. Samples were centrifuged and the pellet washed with 70% ethanol before resuspension in 10 μl RNAse-free water. Libraries were prepared using the TruSeq^®^ Stranded Total RNA Library Prep Kit with Ribo-Zero Globin Set A and B (Illumina^®^, USA) with multiplexing of six samples per lane. Next generation sequencing was performed on the samples using the Illumina^®^ HiSeq^™^ 2500. Base calling was completed using Real Time Analysis software (v1.18.6) (Illumina^®^). Library preparation and sequencing were performed at the Lausanne Genomic Technology Facility (UNIL, Lausanne). The raw and processed RNA data generated from this study have been deposited in NCBI’s Gene Expression Omnibus [29] (GEO Series accession number GSE98645): https://www.ncbi.nlm.nih.gov/geo/query/acc.cgi?acc=GSE98645

### Statistical methods

#### Clinical biomarkers and appetite sensations questionnaire

The postprandial response to each dairy product was evaluated with clinical biochemistry, inflammatory and appetite parameters by linear calculation of the incremental area under the curve (MESS package, v0.3–2 [30]). The postprandial responses were compared using the Wilcoxon signed-rank test (paired). Carryover effects were assessed using a pre-test as described by Welleck and Blettner [31]. Significant effects were considered if *p* ≤ 0.05. All clinical data analysis was completed in R (v3.2.4) [32].

#### Processing of gene expression data

Sequencing data quality was assessed using FastQC (v0.11.4) [33]. Sequences were mapped to the Human Genome (version Hsapiens GRCh37) using the bcBio-nextgen pipeline (v0.9.6a), applying cutadapt (v1.9.1) [34], aligning reads with STAR (v2.5.0c) [35], and counting sequences per gene with featureCount (v1.4.4) [36]. Non protein-coding genes and genes with no assigned counts were removed prior to normalisation. The edgeR package (v3.12.1) [37] was used to calculate normalisation factors for the scaling of raw library sizes applying the ‘TMM’ method that uses the weighted trimmed means of log-ratios (with respect to the reference) [38]. These factors were then used to normalise the data by the voom function [39] (Limma package, v3.26.9 [40]). This process of normalisation was completed in two steps: firstly, for the purpose of identifying genes with low counts (all genes with a mean expression of less than zero after normalisation were removed), secondly, the raw counts were normalised for the dataset with these low count genes excluded. Ensembl IDs were converted to gene symbols using BioMart (v2.26.1, Ensembl Genes 86, Dataset version GRCh37.p13) [41, 42]. The composition of the three main white blood cell types was estimated using CellMix package (v1.6) [43] that applies a deconvolution method using gene expression profiles of blood cell types [44].

#### Gene expression analyses

All gene expression data analysis was completed in R (v3.2.5) [32]. Data exploration was carried out using principal components analysis and hierarchical cluster analysis. Inter-individual variation was detected in this analysis and the methods used for the gene expression analyses were specifically chosen to account for this effect. The postprandial response was thus assessed by calculating, for each subject and each dairy test, the difference in the gene expression profile of the three postprandial measures (2, 4, and 6 h) and the gene expression profile for the corresponding fasting sample, prior to completing correlation or differential analyses [45]. Similarly the short-term effects were assessed by first calculating the difference in fasting gene expression profiles comparing pre- and post-dairy exposures for each subject.

#### Gene expression analyses: Correlation analysis with clinical biomarkers

To assess the relationship between postprandial gene expression in whole blood and the circulating clinical biomarkers (metabolic and inflammatory biomarkers) Spearman’s rank correlation test was used. For each of the three postprandial time points (2, 4, and 6 h), the delta change of the clinical biomarkers (with respect to fasted time, 0 h) was correlated with the respective change in gene expression for each gene assessed. Analyses were only completed for clinical biomarkers that showed a significant response at the selected time point compared to fasting assessment for both dairy products (pooled). This was evaluated by the one-sided Wilcoxon-test. For each correlation analysis, genes were ordered by Spearman’s rank coefficient (rho) and geneset enrichment analysis (GSEA) [46] was carried out for the ‘metabolic’ subset of genesets defined in the Hallmark human reference geneset collection (mSigDB, Broad Institute, v5) [47]. The GSEA method aligns a ranked list of genes to a reference geneset and then calculates an ‘enrichment score’ (ES) using a weighted Kolmogorov Smirnov test to assess the difference in distribution of the reference geneset within the ranked gene list compared to the expected distribution of the geneset if it were uniformly distributed within the list. A ‘normalised’ enrichment score (NES) is calculated to correct for differences in number of genes within each geneset. Significance of the enrichment is assessed by comparison to the ES obtained by random permutation of the ranked gene list, with adjustment for multiple comparisons using the false discovery rate method (*p*_*adj*_) [48]. In our study 100,000 iterations were carried out for each comparison.

#### Gene expression analyses: Differential analysis

Differential analysis was carried out using Limma (v3.26.9 [40]) with empirical Bayes moderation of variance to identify genes that showed a postprandial response during the 6 h following the intake of either dairy product and genes that were modulated by the short-term (two weeks) daily intake of the dairy products. The analysis was completed separately for each dairy product and was also applied to detect differences in the effects of the two dairy products on gene expression. In addition, the stability of gene expression was explored by completing differential analysis to compare the two fasting measurements obtained on the dairy postprandial test (D1 and D2, Fig 1). Inter-individual variation was further controlled for by using the blocking feature within Limma where appropriate. False discovery rate was applied to correct for multiple testing [48]. Further hierarchical analysis using Euclidean distance and Ward’s criterion [49] was carried out to explore the kinetic characteristics of genes that showed a significant postprandial response.

The results of the differential analyses were explored using the GSEA method [46] using the full Hallmark human reference genesets (mSigDB, Broad Institute, v5 [47]) with 100,000 permutations to assess the level of significance (as described for the correlation analyses). Significant genesets were identified as those with a *p*_adj_ < 0.10. The genes that contributed to the significant enrichment of genesets (i.e. the ‘leading edge’ genes) were analysed to assess geneset redundancy and overlaps using hierarchical clustering using Euclidean distance and Ward’s criterion [49]. On the basis of this cluster analysis and the functional grouping defined for the Hallmark collection [47], six groups were defined to resume the genes contributing to the enriched genesets (S2 Table). In addition, GSEA analysis was completed specifically for the KEGG ‘Insulin Signaling Pathway’ geneset to target insulin- related genes (C2: curated geneset, mSigDB, Broad Institute, v5 [47] derived from KEGG database [50–52]).

#### Gene expression analyses: Targeted *AhR* analysis

Metabolomics analysis of the postprandial samples for this study identified four indoles derivatives that were differentially regulated after the intake of acidified milk or yoghurt (indole-3-lactic acid (ILA), indole-3-acetic acid (IAA), indole-3-acetaldehyde (IAAld) and 3-indolepropionic acid (IPA)) (Pimentel *et al*., *submitted*). Of these derivatives, IAA, IAAld and ILA have previously been shown to act as ligands or activate the aryl hydrocarbon receptor (AhR) [53–57]. Therefore, a targeted correlation analysis was completed to explore the relationship between the expression of *AhR* and these four indoles compounds (Spearman’s rank correlation test). In addition, the relationship between the postprandial changes of the expression of *AhR* and the postprandial changes in insulin and glucose were examined using Spearman’s rank correlation test, based on previous data that linked these clinical parameters to altered AhR activation [58–60].

## Results

### Baseline characteristics

The baseline characteristics for the thirteen subjects that were included in the final analyses of this study have previously been reported [21]. The clinical biomarkers and appetite sensations questionnaire data reported here also concern the full study cohort. Gene expression was studied in a subset of seven subjects. As for the main cohort, these subjects were young, healthy and with all fasting biochemical parameters within the normal range at baseline.

### Postprandial responses to dairy intake for clinical biomarkers and appetite sensations

Yoghurt intake induced a significantly greater postprandial insulin response (iAUC) compared with acidified milk whilst no differences between the products were observed for the glycemic response (S3 Table). Analysis of the lipid parameters (triglycerides and cholesterol fractions) showed no differences between the dairy postprandial responses. Little differences were observed for the responses in circulating inflammatory parameters although a non-significant trend towards a lower response after yoghurt compared to acidified milk was noted for TNFα (*p* = 0.10). No significant differences were observed for the responses in hunger, satiety, prospective food consumption, appetite or subjective comfort, comparing the questionnaire responses between acidified milk and yoghurt (S4 Table). While a carryover effect was observed for one appetite parameter, in the absence of an intervention effect this was considered an artifact of the sample size.

### Gene expression data

A total of 12,038 genes were retained for analysis after data filtering. Cellmix predictions of the proportions of the blood cell types (based on cellular signatures for whole blood) [43] showed that the relative proportions of the three main groups of white blood cells were close to the expected proportions for healthy adult populations for all but one sample (fasting sample, post-acidified milk) (S1 Fig). Where appropriate, analyses were completed with and without this data point but the most significant enrichments persisted in the absence of the data point. The largest source of variability in the predicted cell type composition was due to inter-individual variability. However, inter-individual variability was taken into consideration in our analyses in several ways: by the use of a crossover study design, by calculation of changes in gene expression with respect to baseline values, and, where necessary, by introducing subject identity as a blocking factor within the Limma model for assessing differential expression. Postprandial changes in the cellular composition were observed but these were similar between the dairy products for the same subject.

### Clinical biomarker and gene expression correlation analysis

Exploratory correlation analyses were completed to assess the relationship between the postprandial changes in gene expression and selected clinical biomarkers. The clinical biomarkers and time points included in this analysis were glycemia, insulin, IL6, TNFα, CCL5 and LPS at 2h, and triglycerides, total cholesterol, LDL-cholesterol and LPS at 6 h. Enrichment analysis completed for all filtered genes ranked by the strength of their correlation with the target biomarker identified five metabolic genesets that were associated with the biomarkers (Table 1).

**Table 1:** Metabolic genesets enriched when genes are ranked by the correlation coefficient for change in gene expression and change in clinical parameter.

Clinical Parameter	Time clinical parameter assessed (h)	Geneset	Time gene expression assessed (h)	ES	NES	*p*	*p*_adj_
LPS	2	Heme metabolism	2	0.42	1.24	0.01	0.72
LPS	2	Heme metabolism	6	0.41	1.28	0.03	1.00
LPS	6	Heme metabolism	2	0.42	1.39	0.00	0.04*
LPS	6	Heme metabolism	4	0.49	1.61	0.00	0.001*
LPS	6	Heme metabolism	6	0.38	1.22	0.04	1.00
Total cholesterol	6	Heme metabolism	2	0.51	1.67	0.00	0.001
Total cholesterol	6	Cholesterol homeostasis	2	0.45	1.33	0.04	1.00
Total cholesterol	6	Bile acid metabolism	2	-0.43	-1.30	0.05	1.00
Total cholesterol	6	Bile acid metabolism	6	-0.49	-1.45	0.00	0.30
LDL-cholesterol	6	Heme metabolism	2	0.53	1.69	0.00	0.001*
LDL- cholesterol	6	Heme metabolism	6	0.36	1.20	0.04	1.00
Insulin	2	Oxidative phosphorylation	4	-0.41	-1.30	0.01	0.50
Glycemia	2	Fatty acid metabolism	2	0.44	1.30	0.03	1.00
Glycemia	2	Heme metabolism	4	-0.42	-1.25	0.03	1.00
Glycemia	2	Oxidative phosphorylation	4	-0.39	-1.20	0.04	1.00
Glycemia	2	Cholesterol homeostasis	4	0.46	1.31	0.04	1.00
Glycemia	2	Glycolysis	2	0.41	1.23	0.05	1.00
CCL5	2	Oxidative phosphorylation	4	-0.38	-1.23	0.04	1.00

Negative associations are indicated by the minus sign for the peak enrichment score (ES) or for the normalised peak enrichment score (NES). All associations with *p* ≤ 0.05 are shown;

* indicates significant correlations (*p*_adj_ < 0.10).

Abbreviations: CCL5, chemokine ligand 5; ES, enrichment score; LPS, lipopolysaccharide; NES, normalised peak enrichment score.

Positive correlations between clinical parameters and gene expression were notably observed between glycemia and genes of the glycolysis geneset, i.e. genes that were ranked by correlation coefficient for the delta change in glycemia at 2h and change in gene expression at the same time showed a trend towards enrichment of the Hallmark geneset for glycolysis (*p* = 0.05). Glycemia at 2 h was also associated with an enrichment in genes of the fatty acid metabolism geneset at 2 h and with an enrichment of genes in the cholesterol homeostasis geneset at 4 h. Change in LPS levels were strongly correlated with genes of the heme metabolism geneset with significant enrichments for the geneset at multiple time points assessed. Geneset enrichments were also observed for genes that were negatively associated with clinical parameters. Interestingly, oxidative phosphorylation was enriched when genes were ranked by correlation coefficient for insulin, glycemia and CCL5 assessed at 2 h and gene expression assessed at 4 h. Similarly, the bile acid metabolism geneset was enriched for genes ranked by correlation between change in total cholesterol at 6 h and change in gene expression at both 2 and 6 h.

### Postprandial blood transcriptome: General trends

**Fig 2:**
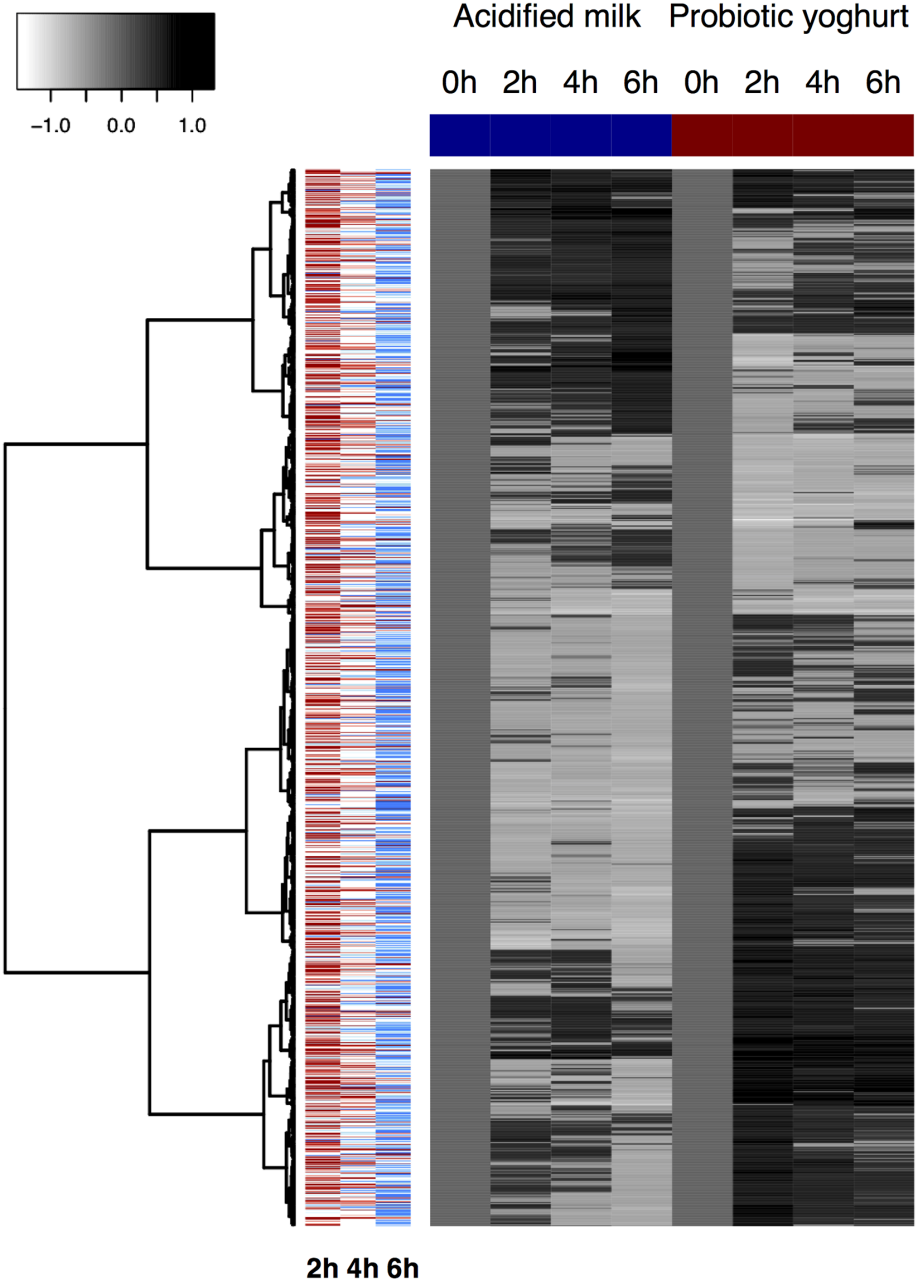
Heatmap showing the dynamic changes in gene expression after the intake of acidified milk (left) and yoghurt (right). Genes that show a postprandial response (*p*_adj_ < 0.20) for at least one time point after either dairy product are visualised (*n* = 1,556), with up-regulation indicated by darker tones relative to fasting levels (0 h) and down-regulation indicated by lighter tones. Clustering was completed using Euclidean distance and Ward’s criterion [49]. For the three times assessed, colours in the left side panel indicate whether the observed response was greater after yoghurt or acidified milk (assessment based on the absolute *t* values: red = response greater after yoghurt, blue = response greater after acidified milk; dark colours show responses significant at a level of *p* < 0.01, light colours indicate responses significant at a level of *p* < 0.05).

The most significant postprandial regulation of individual genes was observed after yoghurt intake; eleven genes were significantly regulated after yoghurt (*p*_adj_ < 0.10) (S5 Table), while no genes were regulated after acidified milk at the same level of significance. A total of 1,556 genes showed a postprandial response (for at least one time point) after either yoghurt (775 genes) or acidified milk intake (832 genes) (*p*_adj_ < 0.20), including 51 (3%) genes that were modulated by both dairy products (Fig 2). The response to yoghurt intake was predominantly observed at 2 h with 747 genes being regulated at this time. In contrast, at 4 and 6 h after yoghurt intake respectively only 55 and 4 genes were different to fasting measurements (*p*_adj_ < 0.20). An inverse trend was observed for the genes that were regulated by acidified milk intake; while no genes were significantly modulated at 2 or 4 h after acidified milk intake, at 6 h the expression of 832 genes was different to fasting measurements (*p*_adj_ < 0.20). Comparison of the postprandial response to yoghurt with that of acidified milk revealed only two genes that were differentially regulated (*p*_adj_ < 0.20).

### Postprandial blood transcriptome: Enriched genesets

Using GSEA, eighteen genesets were found to be significantly enriched after either ingestion of acidified milk and/or yoghurt (*p*_adj_ < 0.10) while trends towards enrichment after the interventions were identified for a further eight genesets (*p*_adj_ < 0.20) (S6 Table). The most significant enrichments (*p*_adj_ < 0.10) were characterised by six groups of genesets (grouping based on the similarity of the genes that were regulated and Hallmark classification, as defined in S2 Table), resumed as ‘immune’, ‘cellular metabolism’, ‘development’, ‘cellular signalling’, ‘heme metabolism’ and ‘other pathways’ (S2 Fig). One of the important groups of genesets to be regulated was ‘cellular metabolism’ that included the ‘glycolysis’ geneset. This geneset was neither modulated by yoghurt nor acidified milk at 2 h, however, it was down-regulated at 4 h after acidified milk intake (*p*_adj_ = 0.05) and at 6 h after yoghurt intake (*p*_adj_ = 0.04). It should be noted that this geneset comprised genes of both glycolysis and gluconeogenesis metabolic pathways (as described by the KEGG pathway database [50–52]) but the two significant negative enrichments observed after the dairy intake concerned both pathways (S3 Fig). The related KEGG insulin signaling pathway was assessed in a targeted manner based on the significant differences observed for postprandial change in circulating insulin between the dairy products. The targeted GSEA revealed a positive enrichment in the pathway after yoghurt intake at 2 h (NES = 1.3, *p* = 0.03) (S4A Fig) and a similar trend after acidified milk at 2 h (NES = 1.2, *p* = 0.10) (S4B Fig).

**Fig 3:**
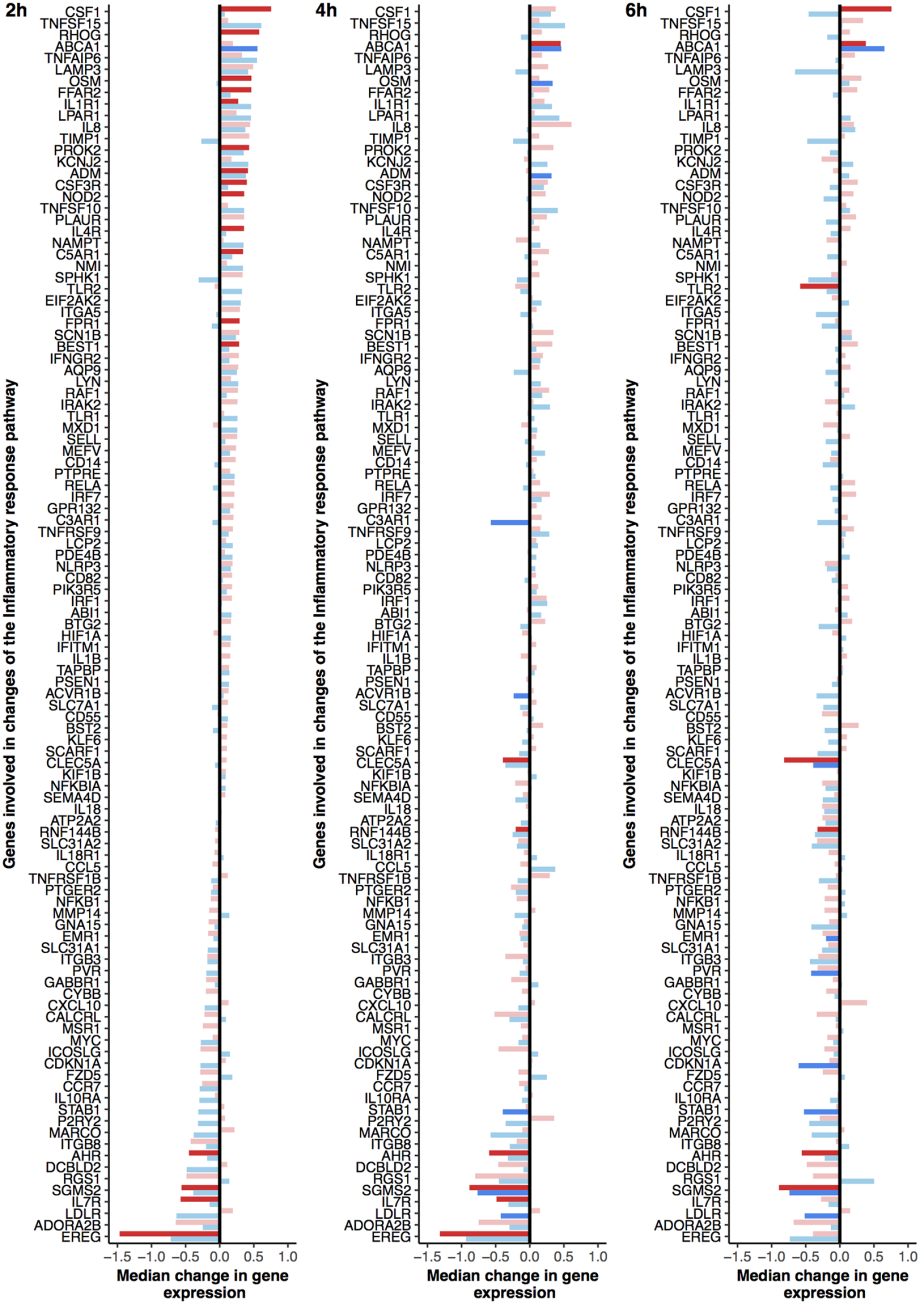
Postprandial changes of genes implicated in the regulation of the inflammatory pathway after intake of yoghurt or acidified milk. The genes contribute to up-regulation of the pathway at 2 h following both dairy products (acidified milk *p*_adj_ = 0.10, yoghurt *p*_adj_ = 0.16), and down-regulation of the pathway at 4 h and 6 h (acidified milk 4 h *p*_adj_ = 0.14 and 6 h *p*_adj_ > 0.20, yoghurt 4 h *p*_adj_ = 0.04 and 6 h *p*_adj_ = 0.07). The median change in gene expression (with respect to fasting levels) is illustrated for each gene by a single bar (red for yoghurt, blue for acidified milk). Lighter colours show non-significant changes as compared to dark shades (*p* < 0.01). Genes are ranked by the greatest change at 2 h to observe the evolution of the postprandial response.

**Fig 4:**
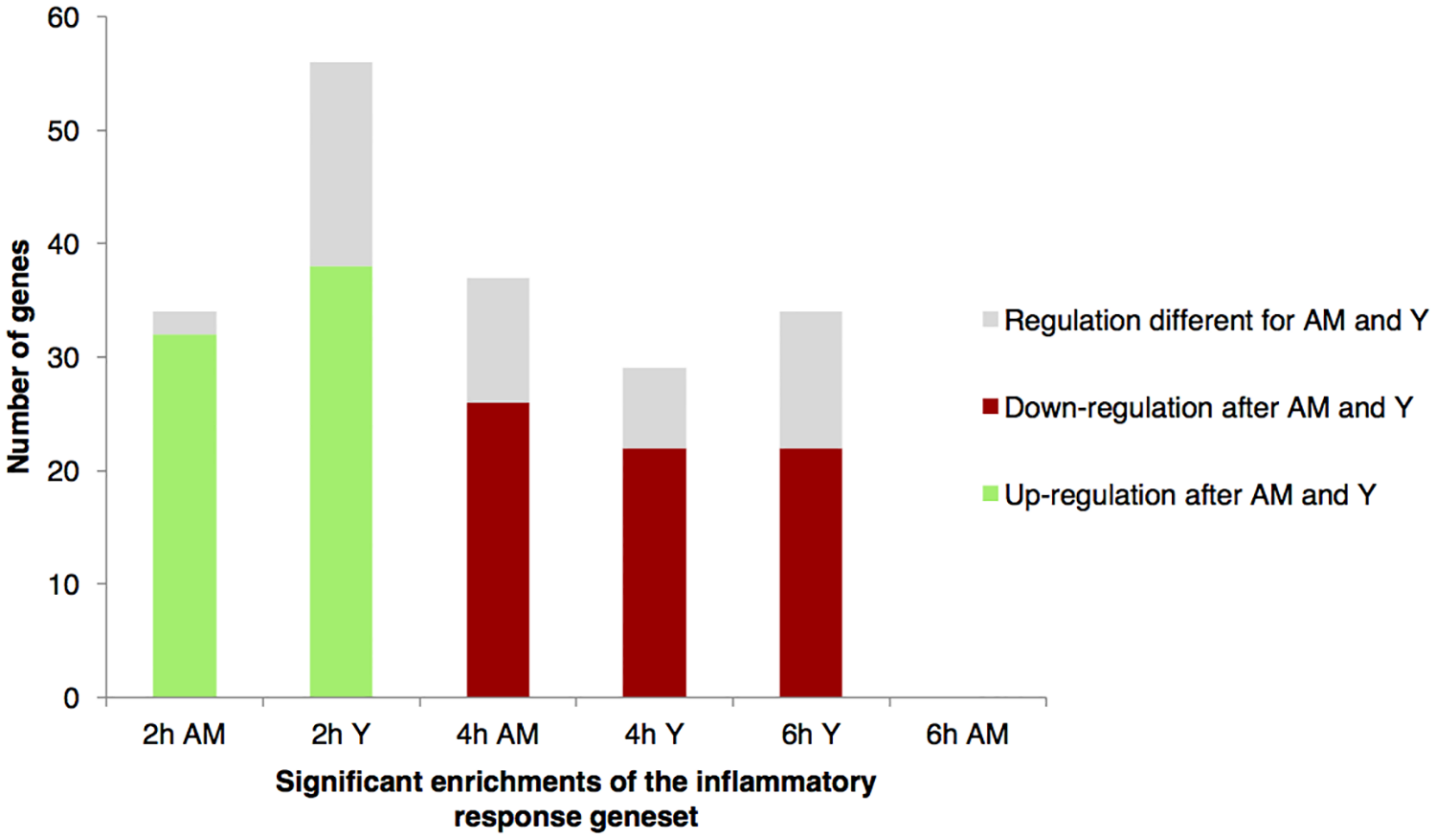
Similarities and differences in the regulation of genes that contribute to enrichments of the inflammatory response geneset after dairy intake. Each bar shows the total number of genes that contributed to the enrichment for the indicated condition (yoghurt or acidified milk; 2, 4 or 6 h postprandially) and the bar is coloured to indicate whether the genes in the enrichment were regulated in the same manner after the alternative dairy product. Abbreviations: AM, acidified milk; Y, yoghurt.

The other major group of genesets that was regulated after both acidified milk and yoghurt intake comprised the immune or inflammatory-related pathways. The significant postprandial changes associated with the immune or inflammatory pathways were broadly characterised by positive enrichments during the early postprandial response (2 to 4 h), however the ‘inflammatory response’ geneset was dynamically regulated as illustrated in Fig 3. The postprandial response of the geneset was characterised by an increase in gene expression at 2 h that was for the most part common to both acidified milk and yoghurt (respectively *p*_adj_ = 0.02 and 0.17), although the yoghurt response included more genes that individually showed a significant postprandial response. Conversely, at 4 h the gene expression of the inflammatory response pathway compared to fasting values showed a relative reduction that was more pronounced after yoghurt intake (*p*_adj_ = 0.02) than after acidified milk intake (*p*_adj_ = 0.20). Genes that were down-regulated at 2 h appeared to be stably expressed or even show increased down-regulation during the late postprandial response, while genes that were up-regulated at 2 h showed a marked reduction in this up-regulation by 6 h. Of note, as illustrated in Fig 4, the direction of change (up- or down-regulation) observed for most of the genes that contributed to the dynamics of this pathway was globally the same after yoghurt and acidified milk intake; in particular, 94% of the genes that were up-regulated after acidified milk at 2 h were also up-regulated after yoghurt at 2 h. These values are notably higher than those calculated for the sum of all postprandially regulated genes (3%).

**Fig 5:**
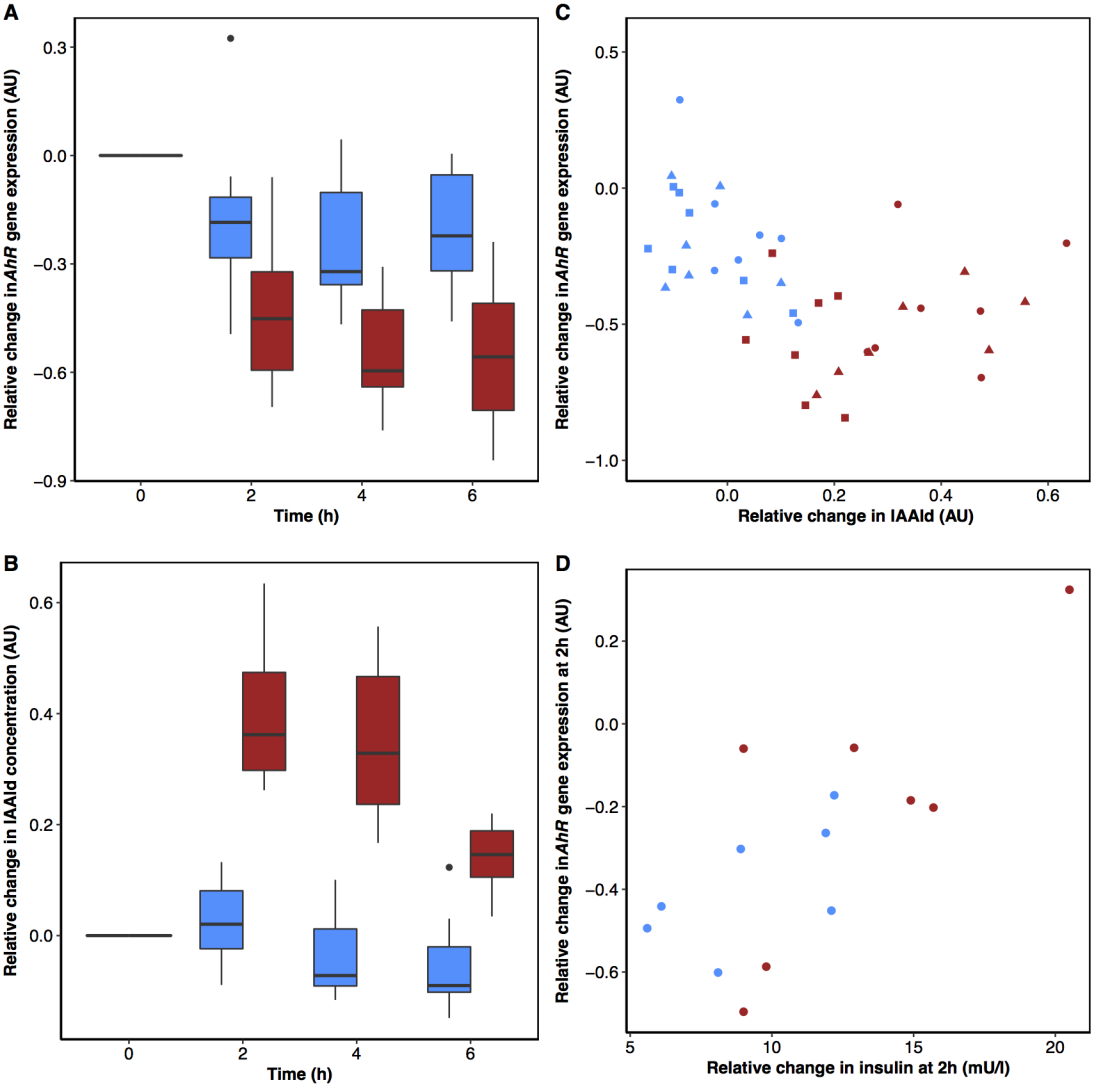
Association between expression of the aryl hydrocarbon receptor (*AhR*) gene in blood cells and circulating concentrations of indole-3-acetaldehyde (IAAld) and insulin. Postprandial changes in *AhR* expression (A) and in circulating concentrations of IAAld (B) (Pimentel *et al*.,*submitted*) following dairy intake. Postprandial changes of *AhR* correlate with IAAld after acidified milk intake (rho = -0.43, *p* = 0.05) but not after yoghurt intake (rho = 0.28, *p* = 0.20) (C). Changes in *AhR* expression at 2 h postprandially correlate with changes in insulin at 2 h after yoghurt intake (rho = 0.75, *p* = 0.05) with a similar trend after acidified milk intake (rho = 0.61, *p* = 0.14) (D). Acidified milk, blue and yoghurt, red. Symbols represent the time of sampling: 2 h (circles), 4 h (triangles) and 6 h (squares). Abbreviations: *AhR*, aryl hydrocarbon receptor; IAAld, indole acetaldehyde.

Two genes that significantly contributed to the negative enrichments of the inflammatory genesets after yoghurt intake were identified as *AhR* and epiregulin (*EREG*), a growth factor regulated by AhR [61–64]. Indeed, both genes were already identified among the top eleven most regulated genes after dairy intake (S5 Table). Independent metabolomics analysis (LC-MS) for this study identified AhR ligands among the metabolites that discriminated the postprandial responses of the dairy products (Pimentel *et al*., *submitted*). Correlation analysis suggested some associations between the postprandial changes in *AhR* expression after dairy intake (Fig 5A) and the postprandial changes in concentration of the AhR ligand, IAAld (Fig 5B) but the relationship did not appear to be linear (Fig 5C). In addition, a positive association between the change in circulating insulin and the change in the expression of *AhR* at 2h after yoghurt intake was observed (rho = 0.75, *p* = 0.05), while a similar trend was observed after acidified milk intake (rho = 0.61, *p* = 0.14) (Fig 5D).

### Postprandial blood transcriptome: Differentially enriched genesets

**Fig 6:**
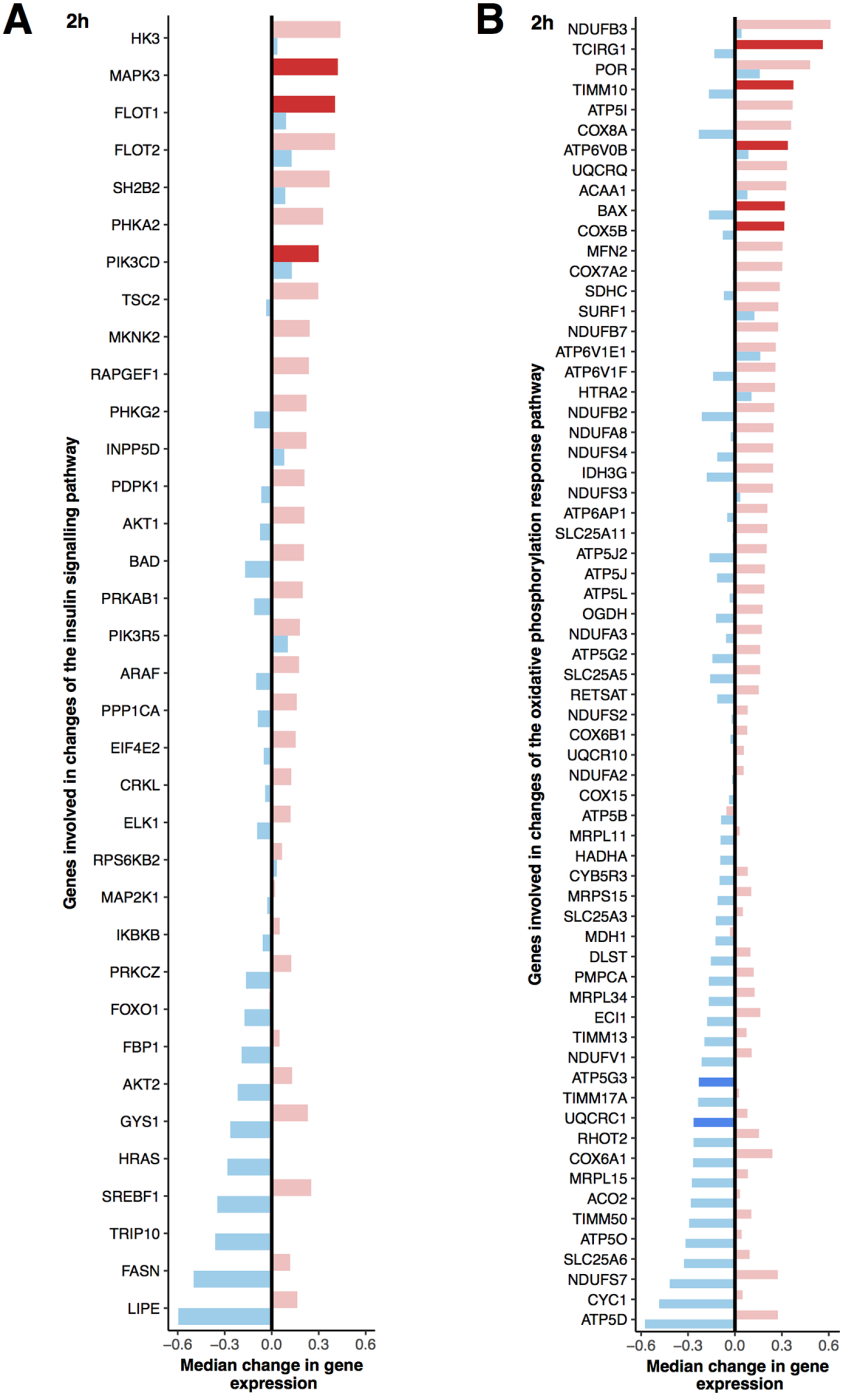
Genes that are implicated in the differential regulation of A. KEGG insulin signaling pathway and B. oxidative phosphorylation pathway at 2 h. The median response for each gene is illustrated by a bar (blue for acidified milk, red for yoghurt) and ordered by the dairy condition that elicited the greatest change in gene expression. Lighter colours show non-significant responses and dark shades show a significant change after intake of the dairy product (*p* < 0.01).

Comparison of the postprandial gene expression changes at 2 h after yoghurt intake to those after acidified milk intake revealed a differential enrichment in the KEGG insulin signaling geneset (NES = 1.4, *p* = 0.01) (Fig 6A). The genes that were differentially expressed in the pathway (S5 Fig) included both genes that were specifically modulated by yoghurt (S4A Fig) and those that were regulated in a different manner after acidified milk intake (S4B Fig).

The comparison of the postprandial gene expression after acidified milk with that of yoghurt intake did not reveal any significant enrichments for the Hallmark genesets. However, a trend towards increased enrichment of IL6 JAK STAT3 signaling (NES = 1.5, *p*_adj_ = 0.24), PI3K AKT MTOR signaling (NES = 1.4, *p*_adj_ = 0.36), and oxidative phosphorylation (NES = 1.2, *p*_adj_ = 0.36) genesets was observed at 2 h after yoghurt intake compared to acidified milk intake, while the MYC targets geneset (version 1) seemed to be enriched after acidified milk intake compared to yoghurt (NES = -1.3, *p*_adj_ = 0.36). The enrichment in oxidative phosphorylation was actually characterised by both a reduction in gene expression after acidified milk and a concurrent increase in expression of genes from the same pathway after yoghurt intake (Fig 6B).

### Blood transcriptome after two weeks daily intake of the test products

Few changes in fasting gene expression were observed after daily intake of the dairy products. Yoghurt intake was associated with a positive enrichment in the MYC targets geneset (version 2) (NES 1.7, *p*_*adj*_ = 0.02) and a negative enrichment of the interferon gamma response geneset (NES -1.5, *p*_*adj*_ = 0.01). Conversely, daily intake of acidified milk was only associated with a negative enrichment of the epithelial mesenchymal transition geneset (NES -1.6, *p*_*adj*_ = 0.02). No differences between the effects of the daily intake of the dairy products on gene expression were observed.

## Discussion

Nutrigenomic tools such as transcriptomics can be used to support the study of the complex consequences of diet on health and disease. In the current study, we used whole blood gene expression together with circulating biomarkers of metabolism and inflammation to explore the physiological effects of probiotic yoghurt compared with non-fermented, acidified milk intake. The two dairy products could be distinguished by the postprandial insulin response which was significantly greater after yoghurt with respect to acidified milk intake. Correspondingly, the insulin signaling pathway was shown to be differently enriched when comparing the responses to the two dairy products at 2 h in our targeted analysis of this pathway, with a positive enrichment in the insulin signaling pathway identified at 2 h after yoghurt intake. Conversely, for both yoghurt and acidified milk intake a similar coordinated regulation of inflammatory or immune-related genes was observed although the timing of the regulation was different.

Our results concur with previous reports of differences in circulating insulin responses to fermented compared to non-fermented milks [7]. In one of our previous transcriptomic studies that applied a similar experimental design [26], we did not observe differences on insulin-related gene expression after intake of a standard yoghurt compared to an acidified milk. The different results in this previous study, may be due to their use of linear contrasts to investigate the direction of change in gene expression between 2 and 6 h rather than the changes at individual time points. In addition, the use of larger volumes of dairy products and presence of the probiotic LGG in the yoghurt used in the current study could have accentuated the different effects of the products on insulin. The mechanisms underpinning the differences we observe for insulin responses in our present work could in part relate to the altered nutrient composition of the yoghurt following fermentation that notably include hydrolysis of lactose by lactic acid bacteria to galactose and glucose, as well as the liberation of insulin-stimulatory peptides and amino acids from milk proteins [4]. Of the amino acids that are released during fermentation, several branched chain amino acids and certain bioactive peptides have known insulinotrophic effects [65, 66]. The characteristics of the dairy matrix are also factors that contribute to differences in the speed of gastric emptying and consequently differences in nutrient uptake between fermented and non-fermented dairy foods [67]. This was minimised in our study by the addition of gluconic acid to milk to mimic pH and consistency of yoghurt. While we did not assess gastric emptying time in this study, the results of our appetite sensations questionnaire showed no differences in the perception of satiety after intake of the two products, suggesting that gastric emptying was not markedly different between the products.

Several energy metabolism pathways that can be influenced by insulin were also modulated by the dairy products. Of note, the postprandial response of genes implicated in the regulation of oxidative phosphorylation was remarkably different at 2 h after acidified milk compared to 2 h after yoghurt intake; we observed an up-regulation of these genes after yoghurt intake and a concurrent down-regulation of most of the same genes after acidified milk intake. As oxidative phosphorylation has previously been proposed as a potential biomarker of the postprandial response [45], we explored the changes in this geneset based on the *p* value of the association rather than on the *p*_*adj*_ value. In contrast to the findings for oxidative phosphorylation, a similar down-regulation was observed for glycolytic genes after both dairy products although this appeared to be earlier after acidified milk. The greater insulin response observed after yoghurt compared to acidified milk, together with the absence of difference in glycemic response may explain the delayed down-regulation in glycolytic genes after yoghurt compared to acidified milk, as insulin stimulates this process [68].

Several genesets that are associated with inflammatory or immune regulation were enriched in the early postprandial response to both acidified milk and yoghurt intake. While the magnitude of up-regulation in inflammatory related genes appeared to be greater after yoghurt intake, the overall enrichment of the geneset was not different between the dairy products. Interestingly the genes implicated in the positive enrichment of inflammatory genesets were generally down-regulated in our study between 4 and 6 h after the dairy intake. The association between dairy products and inflammation is controversial; a recent review suggested that despite the pro-inflammatory effects that dairy products can induce in subjects that are allergic to bovine milk, dairy products may otherwise have anti-inflammatory effects, in particular in individuals with metabolic disorders [20]. The early increase in the expression of inflammatory-related genes in our study is consistent with the transient, physiological postprandial inflammation that is induced by a mixed or fat-rich meal [69, 70]. The subsequent down-regulation of these genes suggests a regulatory control that limits prolonged inflammation. This finding is also in line with our previous work that identified a dynamic response in inflammatory parameters after dairy product intake [26]. The persistent down-regulation of the inflammatory response geneset at 6 h after yoghurt intake in the current study raises the notion that dairy products could impact the inflammatory state beyond the postprandial phase. However, the absence of effects on fasting gene expression after a two-week period of daily intake of the dairy products observed in the current study does not support this hypothesis.

*AhR* and *EREG* (a member of the epidermal growth factor family which is regulated by AhR [61–64]), were identified in this study as genes that were significantly regulated during the postprandial response to probiotic yoghurt and which contribute to multiple inflammatory pathways. The AhR that has a well-defined role in the metabolism of xenobiotics, has recently emerged as a key regulator of inflammatory pathways [71]. Notably, the receptor is described as having a protective role in intestinal cells of the gut upon binding of pseudo-endogenous ligands such as indole derivatives (trypotophan catabolites), which can be produced by the enteric microbiota [54]. As described elsewhere, (Pimentel *et al*., *submitted*) four indole compounds presented a different postprandial response after the intake of the probiotic yoghurt compared to the acidified milk. Among these metabolites, IAAld, a known AhR ligand [54], showed some association with the changes in *AhR* expression following the intake of the dairy products although the relationship was not linear. IAAld was detected in our probiotic yoghurt at higher levels than in the acidified milk and was correspondingly found at higher levels in serum after acute intake of this product compared to acidified milk intake (Pimentel *et al*., *submitted*). The initial decreased expression of *AhR* in the presence of higher levels of the IAAld ligand supports the evidence for an influence of the ligand in the regulation of *AhR* expression [72, 73]. However, at higher concentrations of the metabolite, the modulation of *AhR* expression is less clear. In the context of the distinct differences observed for the postprandial response in insulin together with the previously described role of insulin and glucose on AhR functions [58–60], it was intriguing to find a positive correlation between insulin changes and the change in *AhR* expression at 2 h after the dairy product intake. Conversely, glycemia changes at 2 h were not related to the change in *AhR* expression although the principal glycemic changes in response to the dairy product intakes were observed in the early postprandial phase before 2 h. The effect of insulin sensitivity on AhR ligand activity [59] and the regulatory effect of glucose on AhR activation [58] have important implications for understanding how dietary ligands might interact with the AhR as these parameters are likely to be modulated by a complex meal containing such ligands. It would therefore be interesting to investigate the relationship between IAAld and AhR in the presence or absence of glucose and insulin in a controlled intestinal cell culture model (such as that described by Hubbard *et al*. [54]) in order to better understand the interactions between the nutritional conditions, the ligand activity, and the *AhR* expression.

The validity of the changes in gene expression in our study is supported by their associations with circulating biomarkers. Most notably, the enrichment of the glycolysis geneset for genes that were correlated with glycemia at 2 h showed that genes of the glycolysis pathway were more highly expressed in the presence of higher levels of circulating glucose. Glucose homeostasis is tightly regulated in non-pathological states and thus the close association between glycemia and gene expression of glycolysis, a process that leads to the rapid metabolism of the nutrient, might be expected. Changes in the expression of glycolytic genes in blood during the postprandial phase have previously been identified by Kawakami *et al*., [74] but have not directly been related to glycemia. Changes in some of the circulating parameters that we studied may have direct or indirect effects on gene expression that may not be efficiently modeled by linear associations, however despite this limitation, the method supported the detection of other known associations such as heme metabolism with LPS [75–77] and bile acid metabolism with total cholesterol [78].

The study of the whole blood transcriptome inherently implies a dynamic mixture of cells that will change during a stimuli such as feeding with a known increase in neutrophils and leukocytes postprandially [69, 70]. This was considered in our study by estimations of cellular composition using CellMix and by verifying the consequence of removing a sample identified as an outlier based on estimated cellular composition. While the inter-individual variation in the cell type composition was more marked than the postprandial variation, it should be noted that the observed effects on gene expression that we report, in particular those that are associated with inflammatory processes, may relate to the compositional changes in cell type rather than absolute changes in gene expression. Nevertheless, this approach can still be used as a proxy measure of global gene expression in blood.

The lack of strong effects detected on gene expression during the short-term test phases may be due to other factors (external to diet) that influence the fasting levels of gene expression meaning that the effect of relatively small changes in the diet is difficult to discern. Alternatively, a longer exposure to the dietary intervention used in our study may be necessary to observe an effect on the fasting state. In both cases, the rationale to use the postprandial approach as a sensitive method for assessing diet-specific effects on the blood transcriptome is justified.

## Conclusions

We observed that while probiotic yoghurt and acidified milk elicit different responses on gene expression, this in part relates to differences in the timing of the postprandial response. The dairy products modulated some common pathways during the postprandial phase, notably including various inflammatory or immune genes. The whole blood transcriptome appeared to be a sensitive surrogate marker of metabolic processes that showed coherence with established clinical biomarkers but also revealed subtle differences in the responses to the dairy products that were not captured by these biomarkers. The development of nutrition-specific genesets as well as the combined assessment of metabolomic with transcriptomic data could help better exploit this tool in the context of nutritional interventions. This approach nevertheless remains a promising method to complement classical biomarkers and metabolome evaluation to help better discriminate the metabolic responses to dietary intake in a healthy population.

## Supporting information

S1 Fig Kinetic changes in estimated cell populations for the three major blood cell populations: neutrophils (A), lymphocytes (B) and monocytes (C) for samples taken at during acute intake of dairy (0–6 h) and after 2 weeks daily intake of the dairy products. Kinetics are coloured for each subject (F3_0XX), with symbols representing acidified milk (circles) and yoghurt (triangles) test days. The typical values for neutrophils (green), lymphocytes (red) and monocytes (blue) observed in a healthy population are shown by dashed horizontal lines. The kinetic responses for the three cell types are also shown for each subject separately (D) with darker colours representing acidified milk test days.
(PDF)

S2 Fig Genes that contribute to the significant enrichment of GSEA pathways after acidified milk (AM) or yoghurt intake (Y) at 2, 4 or 6 h postprandially. Grouping based on Hallmark classifications of pathways (Liberzon *et al*., 2015 [47]) and hierarchical cluster analysis of the genes that contribute to significant pathway enrichments (S2 Table).
(PDF)

S3 Fig Changes in gene expression in the glycolysis/gluconeogenesis pathway (KEGG [50–52]) (A) 4 h after intake of acidified milk and (B) 6 h after intake of yoghurt. Gene colours correspond to the *t*-statistic for the Limma assessment of the postprandial responses. Genes that were not detected in the filtered dataset are not coloured.
(PDF)

S4 Fig Gene expression changes in the insulin signaling pathway (KEGG [50–52]) 2 h after intake of (A) yoghurt and (B) acidified milk. Gene colours correspond to the *t*-statistic for the Limma assessment of the postprandial responses at 2h compared to fasting measurements for the each test day. Genes that were not detected in the filtered dataset are not coloured.
(PDF)

S5 Fig Gene expression changes in the insulin signaling pathway (KEGG [50–52]) 2 h after intake of yoghurt compared to acidified milk. Gene colours correspond to the *t*-statistic for the Limma assessment of the differential postprandial response 2 h following intake of yoghurt compared to acidified milk. Genes that were not detected in the filtered dataset are not coloured.
(PDF)

S1 Table Appetite sensations questionnaire (adapted from Flint *et al*., 2000 [27]). (PDF)

S2 Table Classification of genesets into six groups based on both hierarchical analysis of genes that contributed to the enrichments of genesets after yoghurt or acidified milk intake, and functional grouping defined for the Hallmark collection (Liberzon *et al*., 2015 [47]). (PDF)

S3 Table Postprandial response to dairy products assessed for clinical biochemistry and inflammatory parameters. Median response as assessed by the incremental area under the curve compared by crossover analysis as described by Wellek and Blettner (2012) [31], using the Wilcoxon signed-rank test to evaluate significant effects (*p < 0.05). Abbreviations: iAUC, incremental area under the curve; IQR, interquartile range; LPS, lipopolysaccharide; CCL2, chemokine ligand 2; CCL5, chemokine ligand 5; IL, interleukin; TNFα, tumor necrosis factor alpha.
(PDF)

S4 Table Postprandial evaluation of dairy products assessed by a visual analogue scale questionnaire. Median response as assessed by the incremental area under the curve compared by crossover analysis as described by Wellek and Blettner (2012) [31], using Wilcoxon signed-rank test to evaluate the significant effects (*p < 0.05). Abbreviations: iAUC, incremental area under the curve; IQR, interquartile range.
(PDF)

S5 Table Genes that showed a significant postprandial change after yoghurt intake (with respect to fasting values) (analysis completed with Limma [40]). Genes associated with inflammatory pathways in bold.
(PDF)

S6 Table Pathways enriched during the postprandial response after acidified milk or yoghurt intake as assessed by GSEA for relative changes at 2, 4 and 6 h (compared to fasting time 0 h) for each product. * = significant change (*p*_adj_ ≤ 0.10). Trends are shown for *p*_adj_ ≤ 0.20. Direction of regulation is indicated by the enrichment scores (ES): positive values signify up-regulation of the pathway while negative values signify down-regulation of the pathway. Abbreviations: ES, enrichment score; GSEA, geneset enrichment analysis; IL, interleukin; JAK, Janus-family tyrosine kinase; KRAS, KRAS proto-oncogene, GTPase; mTORC1, mammalian target of rapamycincomplex 1; NES normalised enrichment score; NF-kB, nuclear factor kappa-light-chain-enhancer of activated B cells; STAT, signal transducer and activator of transcription; TNFα, tumor necrosis factor alpha; UV, ultraviolet.
(PDF)
