# Supplementary figures and images for: Modulation of the peripheral blood transcriptome by the ingestion of probiotic yoghurt and acidified milk in healthy, young men
Source: PLoS One. 2018 Feb 28;13(2):e0192947. doi: 10.1371/journal.pone.0192947 (PMC5831037; doi:10.1371/journal.pone.0192947)

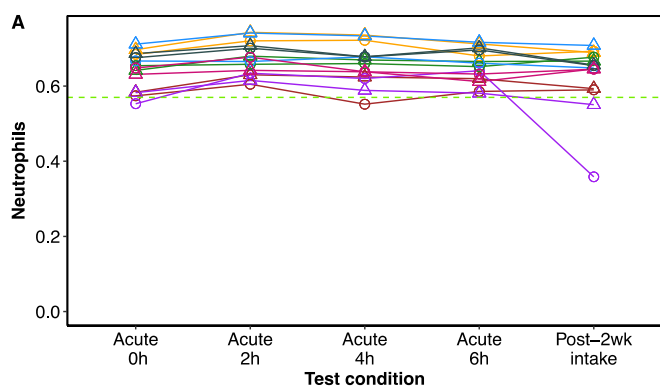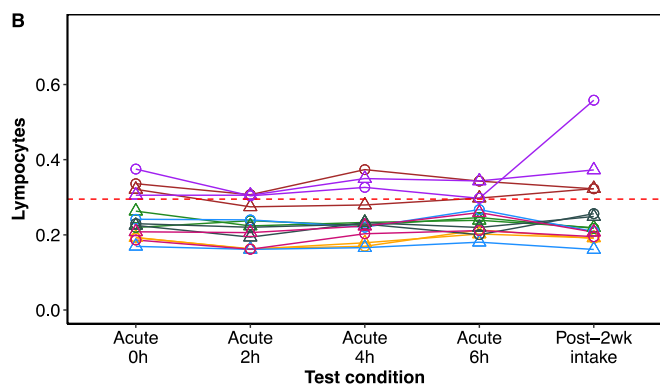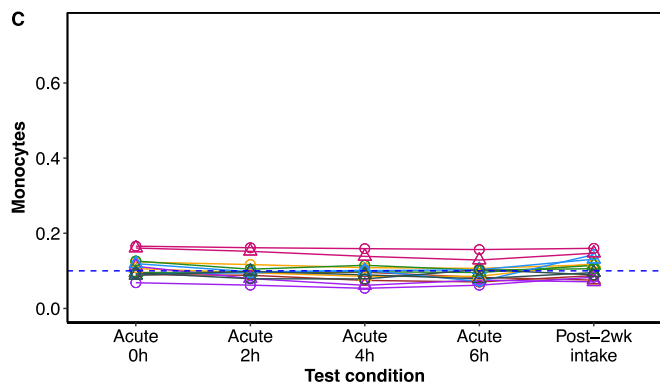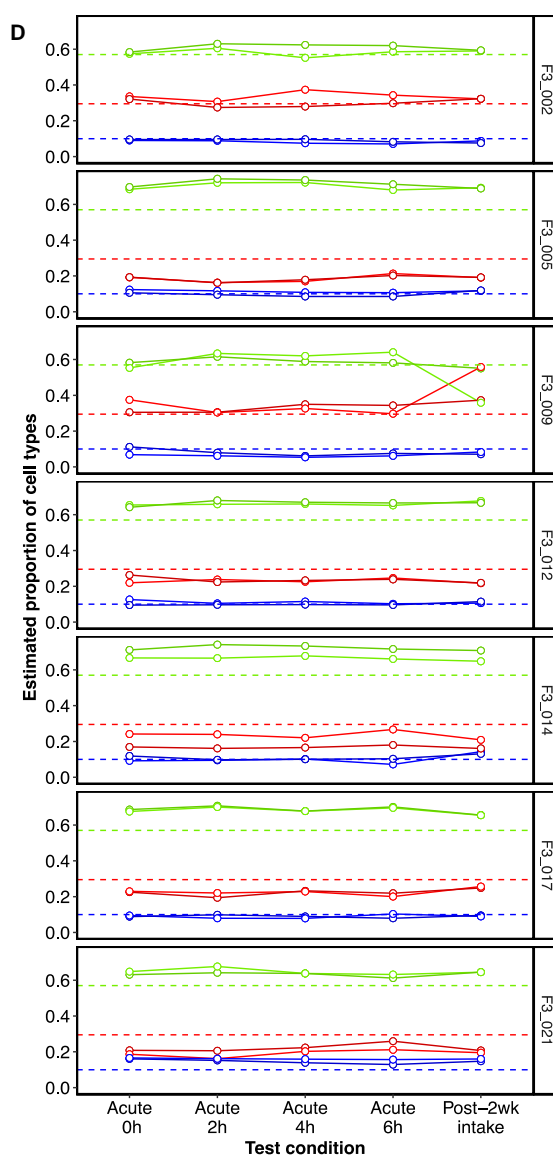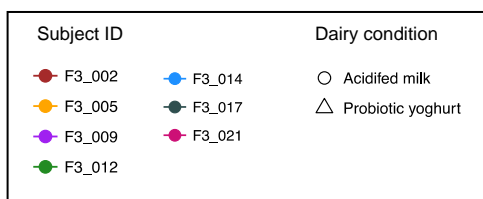

Supplement: S1 Fig — Kinetics are coloured for each subject (F3_0XX), with symbols representing acidified milk (circles) and yoghurt (triangles) test days. The typical values for neutrophils (green), lymphocytes (red) and monocytes (blue) observed in a healthy population are shown by dashed horizontal lines. The kinetic responses for the three cell types are also shown for each subject separately (D) with darker colours representing acidified milk test days. (PDF) [file pone.0192947.s001.pdf]

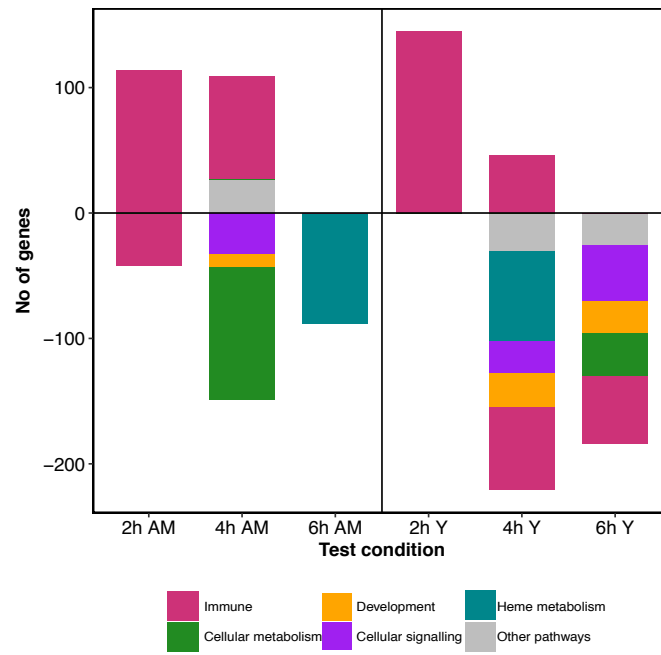

Supplement: S2 Fig — Grouping based on Hallmark classifications of pathways (Liberzon et al., 2015 [47]) and hierarchical cluster analysis of the genes that contribute to significant pathway enrichments (S2 Table). (PDF) [file pone.0192947.s002.pdf]

**A**

GLYCOLYSIS / GLUCONEOGENESIS

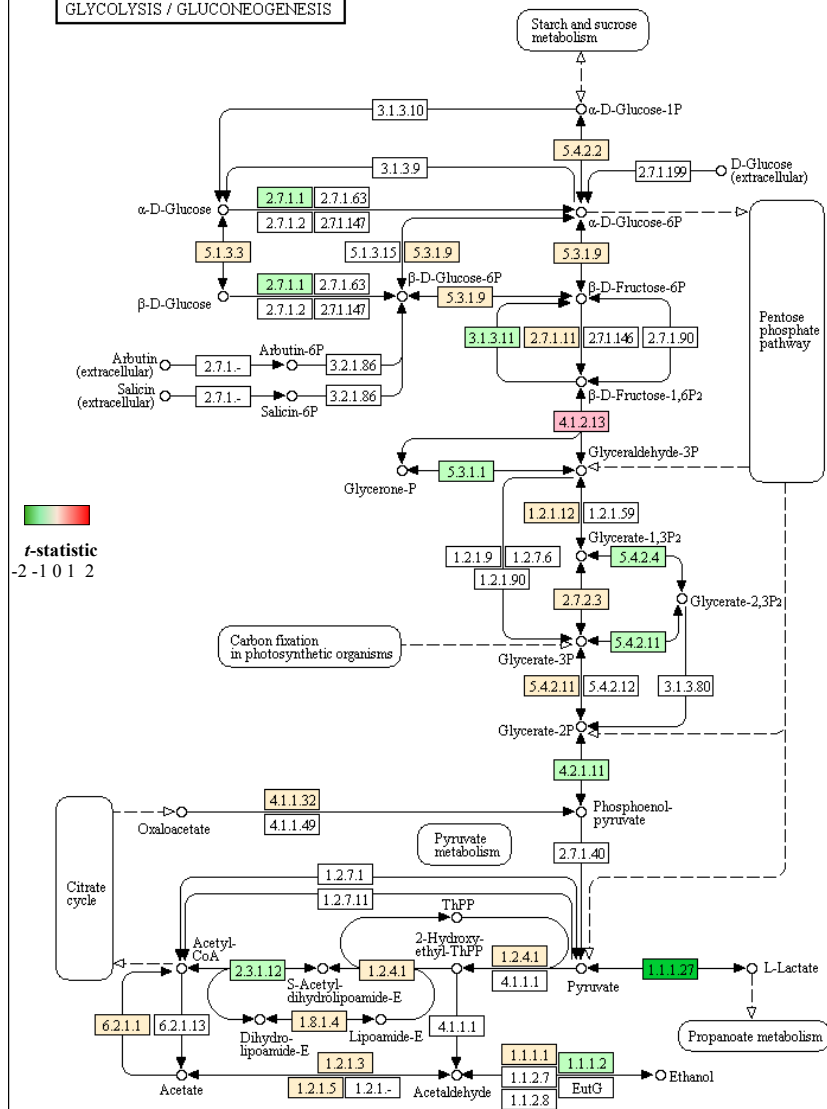

**B**

GLYCOLYSIS / GLUCONEOGENESIS

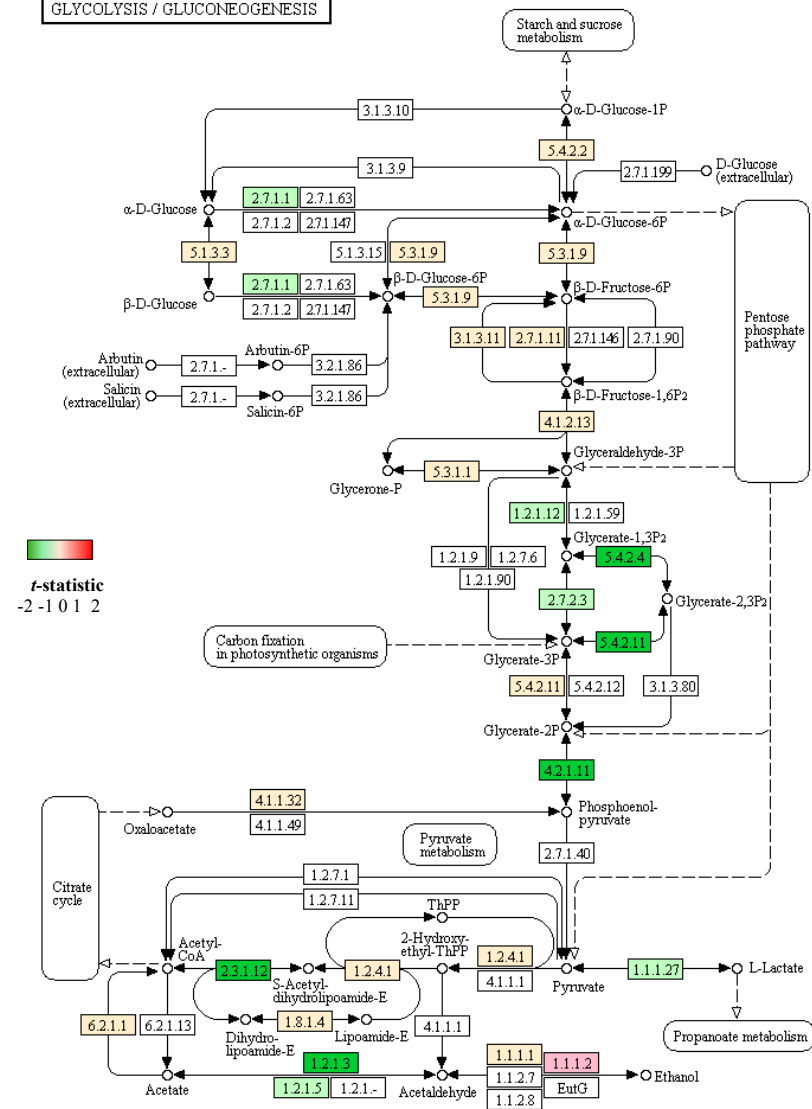

Supplement: S3 Fig — Gene colours correspond to the t-statistic for the Limma assessment of the postprandial responses. Genes that were not detected in the filtered dataset are not coloured. (PDF) [file pone.0192947.s003.pdf]

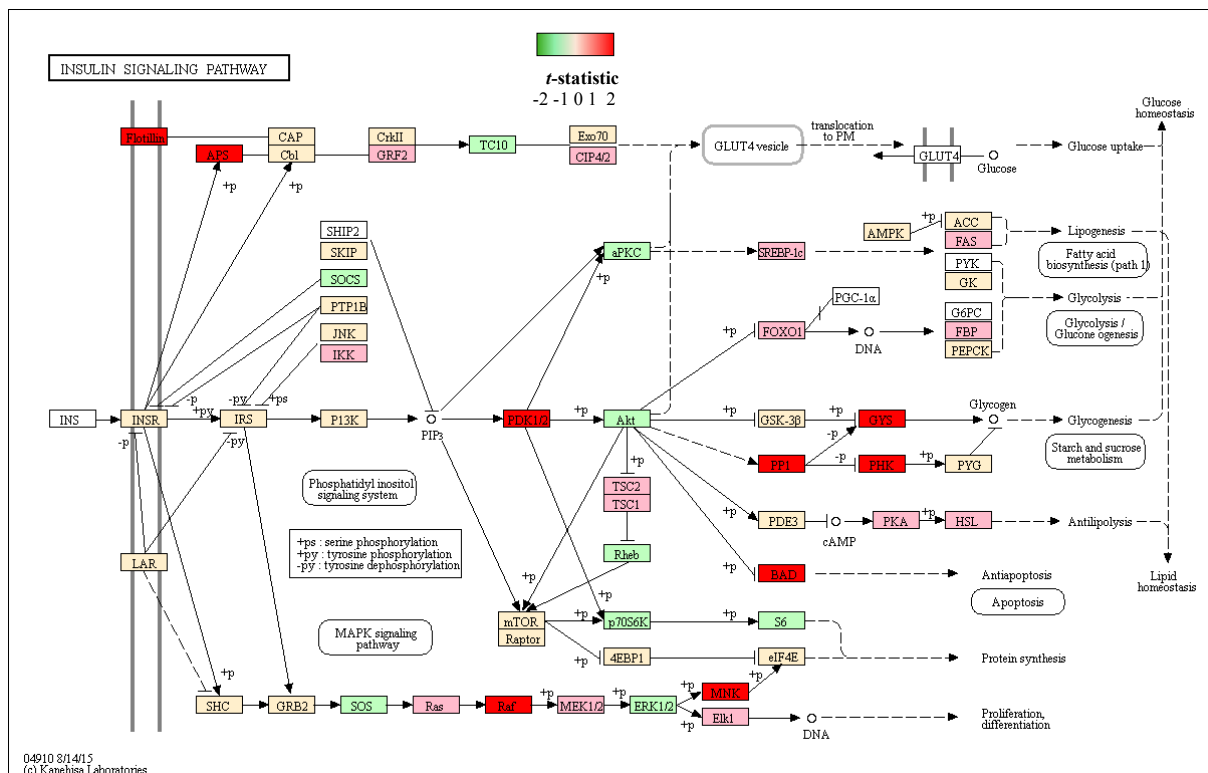

Supplement: S5 Fig — Gene colours correspond to the t-statistic for the Limma assessment of the differential postprandial response 2 h following intake of yoghurt compared to acidified milk. Genes that were not detected in the filtered dataset are not coloured. (PDF) [file pone.0192947.s005.pdf]
