# Supplementary material for: Modulation of the peripheral blood transcriptome by the ingestion of probiotic yoghurt and acidified milk in healthy, young men
Source: PLoS One. 2018 Feb 28;13(2):e0192947. doi: 10.1371/journal.pone.0192947 (PMC5831037; doi:10.1371/journal.pone.0192947)
Supplement: S1 Table — (PDF) [file pone.0192947.s006.pdf]

Question	Assessment	Low response (0)	High response (100)
Q1. How hungry are you?	Hunger	Not hungry at all	Extremely hungry
Q2. How satiated are you?	Satiety	Not at all satiated	Extremely satiated
Q3. How much food do you feel able to eat?	Prospective food consumption	An insignificant amount	A huge amount
Q4. How much do you want to eat savoury foods?	Appetite for savoury foods	Not at all	A lot
Q5. How much do you want to eat sweet food?	Appetite for sweet foods	Not at all	A lot
Q6. How relaxed are you?	State of ease	Not at all relaxed	Extremely relaxed
