# Supplementary material for: Modulation of the peripheral blood transcriptome by the ingestion of probiotic yoghurt and acidified milk in healthy, young men
Source: PLoS One. 2018 Feb 28;13(2):e0192947. doi: 10.1371/journal.pone.0192947 (PMC5831037; doi:10.1371/journal.pone.0192947)
Supplement: S2 Table — (PDF) [file pone.0192947.s007.pdf]

Immune	Cellular metabolism	Development	Cellular signalling	Heme metabolism	Other Pathways
Inflammatory response	MTORC1 signalling	Angiogenesis	Oestrogen response (early)	Heme metabolism	Xenobiotic Metabolism
Interferon gamma response	Glycolysis	Epithelial mesenchymal transition	IL2 STAT5 signalling		Apoptosis
TNFα signalling via NFKB	Hypoxia				UV response (down)
Allograft rejection					
Interferon alpha response					
IL6 JAK STAT3 signalling					
