# Supplementary material for: Modulation of the peripheral blood transcriptome by the ingestion of probiotic yoghurt and acidified milk in healthy, young men
Source: PLoS One. 2018 Feb 28;13(2):e0192947. doi: 10.1371/journal.pone.0192947 (PMC5831037; doi:10.1371/journal.pone.0192947)
Supplement: S3 Table — Median response as assessed by the incremental area under the curve compared by crossover analysis as described by Wellek and Blettner (2012) [31], using the Wilcoxon signed-rank test to evaluate significant effects (*p < 0.05). Abbreviations: iAUC, incremental area under the curve; IQR, interquartile range; LPS, lipopolysaccharide; CCL2, chemokine ligand 2; CCL5, chemokine ligand 5; IL, interleukin; TNFα, tumor necrosis factor alpha. (PDF) [file pone.0192947.s008.pdf]

	Group acidified milk – probiotic yoghurt			Group probiotic yoghurt - acidified milk			Statistical Assessments	
**Parameter**	***n***	**Median iAUC milk (IQR)**	**Median iAUC probiotic yoghurt (IQR)**	***n***	**Median iAUC milk (IQR)**	**Median iAUC probiotic yoghurt (IQR)**	**Carry over effect Sum *P***	**Intervention effect *P***
Glycemia (mmol/l)	6	0.8(-17.8- 16.5)	5.6(-9.9-24.6)	7	-21.0(-28.5- -6)	-25.5(-35.3- 6)	0.174	0.431
Insulin (mU/l)	6	1079.6(897.9- 1275.4)	1552.5(1504.7-1906.3)	7	1004.3(876.4- 1435.9)	1839.8(1363.9- 2170.1)	0.945	**0.001***
Total cholesterol (mmol/l)	6	69.0(27.4- 101.6)	25.5(-12.4-64.5)	7	70.5(60.0- 87.0)	70.5(60- 111.8)	0.138	0.295
HDL cholesterol (mmol/l)	6	9.7(0.4- 18)	-8.3(-16.5-1.1)	7	18.0(9.7- 18.0)	18.0(0.0- 26.3)	0.097	0.612
LDL cholesterol (mmol/l)	6	60.0(39.8- 70.1)	25.5(-21.8-59.3)	7	51.0(24.8- 86.2)	67.5(35.3- 120.0)	0.223	0.534
TAG (mmol/l)	6	57.0(41.6- 85.9)	96.0(79.9-102.0)	7	43.5(34.5- 96.8)	82.5(57.8- 107.3)	0.945	0.295
LPS(EU/ml)	6	237.9(117.8- 348.2)	64.8(8.1-107.1)	7	157.8(103.8- 188.7)	212.4(93.3- 341.7)	0.445	0.534
CCL2 (pg/ml)	6	-44.0(-654.6- 103.9)	-239.9(-593.5-99.7)	7	-821.4(-1300.4- -427.6)	-651.0(-1154.1- -371.8)	0.051	0.445
CCL5(pg/ml)	6	257152.5(237221.3- 327708.8)	39397.5(21622.5-56880.0)	7	161640.0(78900.0- 341080.5)	109845.0(57525.0- 717172.5)	1.000	0.234
IL6 (pg/ml)	6	6926.6(3507.5- 9516.5)	976.7(556.8-1492.5)	7	4894.5(2236.1- 7192.4)	1744.4(835.4- 16501.1)	0.628	0.181
TNFα (pg/ml)	6	4318.2(3586.8- 7347.4)	826.4(544.0-1431.0)	7	4306.4(992.9- 6467.8)	1831.1(769.4- 12597.3)	0.836	0.101
