# Supplementary material for: Modulation of the peripheral blood transcriptome by the ingestion of probiotic yoghurt and acidified milk in healthy, young men
Source: PLoS One. 2018 Feb 28;13(2):e0192947. doi: 10.1371/journal.pone.0192947 (PMC5831037; doi:10.1371/journal.pone.0192947)
Supplement: S4 Table — Median response as assessed by the incremental area under the curve compared by crossover analysis as described by Wellek and Blettner (2012) [31], using Wilcoxon signed-rank test to evaluate the significant effects (*p < 0.05). Abbreviations: iAUC, incremental area under the curve; IQR, interquartile range. (PDF) [file pone.0192947.s009.pdf]

	Group acidified milk - probiotic yoghurt			Group probiotic yoghurt - acidified milk			Statistical Assessments	
**Satiety Questions**	***n***	**Median iAUC acidified milk (IQR)**	**Median iAUC probiotic yoghurt (IQR)**	***n***	**Median iAUC acidified milk (IQR)**	**Median iAUC probiotic yoghurt (IQR)**	**Carry-over effect *P***	**Intervention effect *P***
Hunger	6	-12020(-21072- -4478)	-12254(-15698- -3884)	5	-2704(-3189- -2519)	-341(-3937- 259)	0.329	0.247
Satiety	6	9394(2484- 24404)	17480(6079- 19741)	5	7530(-693- 8293)	148(-1962- 5319)	0.537	0.177
Prospective food consumption	6	-5344(-13456- -1120)	-12951(-16932- -3836)	5	-5758(-7367- 31)	-2769(-4180- -1582)	0.662	0.931
Appetite for savoury foods	6	3181(-1794- 5111)	796(-5494- 4911)	5	1116(284- 4724)	3019(1869- 4451)	0.931	0.792
Appetite for sweet foods	6	-11982(-18801- -2862)	-13600(-17660- -13045)	5	2419(-5953- 4333)	2664(-754- 3589)	**0.009***	0.537
State of ease	6	3365(231- 6331)	6711(3078- 9372)	5	-88(-683- 2965)	3626(157- 3889)	0.247	0.537
