# Supplementary material for: Modulation of the peripheral blood transcriptome by the ingestion of probiotic yoghurt and acidified milk in healthy, young men
Source: PLoS One. 2018 Feb 28;13(2):e0192947. doi: 10.1371/journal.pone.0192947 (PMC5831037; doi:10.1371/journal.pone.0192947)
Supplement: S5 Table — Genes associated with inflammatory pathways in bold. (PDF) [file pone.0192947.s010.pdf]

Ensembl ID	Gene Symbol	Gene name	Time of assessment (h)	*t*-statistic	p_{adj}
ENSG00000171451	DSEL	Dermatan sulfate epimerase-like	2	-8.4	0.07
ENSG00000101460	MAP1LC3A	Microtubule associated protein 1 light chain 3 alpha	2	8.2	0.07
**ENSG00000124882**	**EREG**	**Epiregulin**	**2**	**-7.6**	**0.10**
ENSG00000181019	NQO1	NAD(P)H quinone dehydrogenase 1	2	-7.1	0.10
ENSG00000185875	THNSL1	Threonine synthase like 1	2	-7.0	0.10
**ENSG00000164023**	**SGMS2**	**Sphingomyelin synthase 2**	**4**	**-13.0**	**0.003**
ENSG00000171451	DSEL	Dermatan sulfate epimerase-like	4	-8.7	0.05
**ENSG00000106546**	**AHR**	**Aryl hydrocarbon receptor**	**4**	**-7.1**	**0.10**
ENSG00000126453	BCL2L12	BCL2 like 12	4	7.0	0.10
ENSG00000141384	TAF4B	TATA-box binding protein associated factor 4b	4	-7.0	0.10
**ENSG00000164023**	**SGMS2**	**Sphingomyelin synthase 2**	**6**	**-10.0**	**0.04**
ENSG00000096060	FKBP5	FK506 binding protein 5	6	-8.4	0.08
ENSG00000174776	WDR49	WD repeat domain 49	6	-7.9	0.09
