# Supplementary material for: Modulation of the peripheral blood transcriptome by the ingestion of probiotic yoghurt and acidified milk in healthy, young men
Source: PLoS One. 2018 Feb 28;13(2):e0192947. doi: 10.1371/journal.pone.0192947 (PMC5831037; doi:10.1371/journal.pone.0192947)
Supplement: S4 Fig — Gene colours correspond to the t-statistic for the Limma assessment of the postprandial responses at 2h compared to fasting measurements for the each test day. Genes that were not detected in the filtered dataset are not coloured. (PDF) [file pone.0192947.s004.pdf]

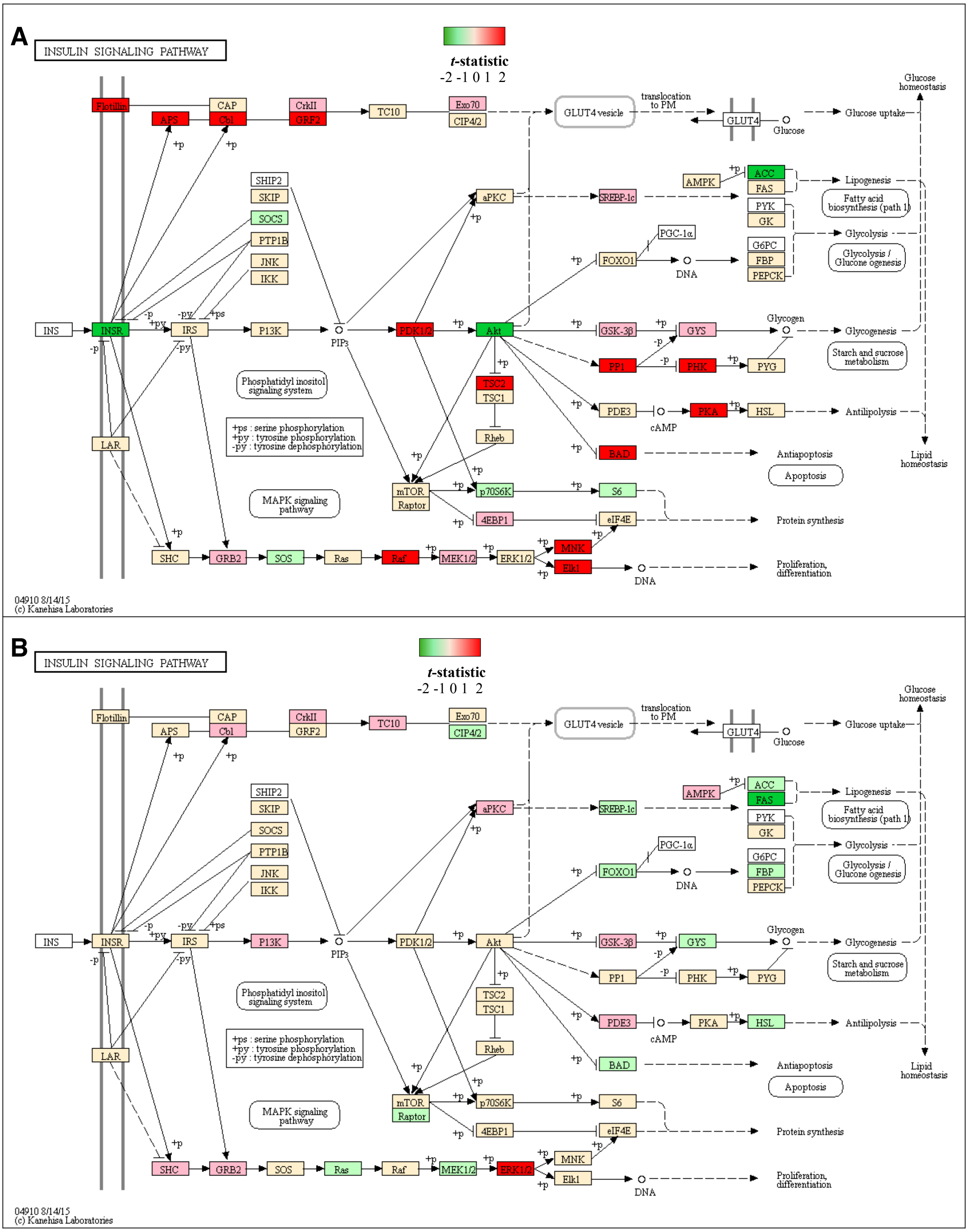
A
INSULIN SIGNALING PATHWAY
t-statistic
-2 -1 0 1 2
Flotillin
CAP
CrkII
TC10
Exo70
GLUT4 vesicle
translocation to PM
GLUT4
Glucose
Glucose uptake
Glucose homeostasis
APS
Cbl
GRF2
CIP4/2
+p
SHIP2
SKIP
SOCS
PTP1B
JNK
IKK
aPKC
SREBP-1c
AMPK
ACC
FAS
PYK
GK
Lipogenesis
Fatty acid biosynthesis (path 1)
PGC-1α
FOXO1
DNA
G6PC
FBP
PEPCK
Glycolysis
Glycolysis / Gluconeogenesis
INS
INSR
IRS
PI3K
PIP3
PDK1/2
Akt
GSK-3β
GYS
Glycogen
Glycogenesis
PP1
PHK
PYG
Starch and sucrose metabolism
-p
+py
-py
+ps
Phosphatidyl inositol signaling system
TSC2
TSC1
Rheb
PDE3
cAMP
PKA
HSL
Antilipolysis
Lipid homeostasis
+ps : serine phosphorylation
+py : tyrosine phosphorylation
-py : tyrosine dephosphorylation
LAR
BAD
Antiapoptosis
Apoptosis
MAPK signaling pathway
mTOR
Raptor
p70S6K
S6
4EBP1
eIF4E
Protein synthesis
SHC
GRB2
SOS
Ras
Raf
MEK1/2
ERK1/2
MNK
Elk1
Proliferation, differentiation
04910 8/14/15
(c) Kanehisa Laboratories
B
INSULIN SIGNALING PATHWAY
t-statistic
-2 -1 0 1 2
04910 8/14/15
(c) Kanehisa Laboratories
