# Supplementary material for: Modulation of the peripheral blood transcriptome by the ingestion of probiotic yoghurt and acidified milk in healthy, young men
Source: PLoS One. 2018 Feb 28;13(2):e0192947. doi: 10.1371/journal.pone.0192947 (PMC5831037; doi:10.1371/journal.pone.0192947)
Supplement: S6 Table — * = significant change (padj ≤ 0.10). Trends are shown for padj ≤ 0.20. Direction of regulation is indicated by the enrichment scores (ES): positive values signify up-regulation of the pathway while negative values signify down-regulation of the pathway. Abbreviations: ES, enrichment score; GSEA, geneset enrichment analysis; IL, interleukin; JAK, Janus-family tyrosine kinase; KRAS, KRAS proto-oncogene, GTPase; mTORC1, mammalian target of rapamycincomplex 1; NES normalised enrichment score; NF-kB, nuclear factor kappa-light-chain-enhancer of activated B cells; STAT, signal transducer and activator of transcription; TNFα, tumor necrosis factor alpha; UV, ultraviolet. (PDF) [file pone.0192947.s011.pdf]

Postprandial responses at 2h			
**Geneset**	**ES**	**NES**	**p_{adj}**
Acidified milk			
Inflammatory response	0.50	1.50	0.02*
Interferon gamma response	0.43	1.34	0.08*
TNFα signalling via NF-kB	0.45	1.38	0.08*
Allograft rejection	-0.43	-1.40	0.08*
TNFα signalling via NF-kB	-0.42	-1.36	0.17
Probiotic yoghurt			
IL6 JAK STAT3 signalling	0.57	1.60	0.009*
TNFα signalling via NF-kB	0.48	1.42	0.01*
Interferon gamma response	0.44	1.32	0.05*
Inflammatory response	0.45	1.31	0.17
Heme metabolism	-0.42	-1.27	0.18
Reactive oxygen species pathway	-0.63	-1.54	0.18
P53 pathway	0.43	1.27	0.18
Androgen response	-0.48	-1.36	0.18
Interferon alpha response	0.44	1.28	0.18
Hedgehog signalling	-0.64	-1.53	0.18

Postprandial responses at 4h			
**Geneset**	**ES**	**NES**	**p_{adj}**
Acidified milk			
Interferon gamma response	0.50	1.48	0.01*
Interferon alpha response	0.54	1.53	0.02*
Apoptosis	0.51	1.47	0.03*
Glycolysis	-0.48	-1.39	0.05*
MTORC1 signalling	-0.45	-1.34	0.05*
Oestrogen response (early)	-0.48	-1.39	0.05*
Hypoxia	-0.46	-1.35	0.10*
Angiogenesis	-0.65	-1.58	0.10*
Inflammatory response	-0.45	-1.30	0.19
Myogenesis	0.47	1.34	0.19
Probiotic yoghurt			
Heme metabolism	-0.54	-1.55	0.001*
Oestrogen response (late)	-0.55	-1.51	0.01*
IL6 JAK STAT3 signalling	-0.65	-1.66	0.01*
Angiogenesis	-0.82	-1.79	0.01*
Inflammatory response	-0.53	-1.46	0.02*
TNFα signalling via NF-kB	0.48	1.40	0.02*
Epithelial mesenchymal transition	-0.54	-1.46	0.03*
UV response (down)	-0.49	-1.35	0.10*
TNFα signalling via NF-kB	-0.48	-1.34	0.10*
P53 pathway	-0.46	-1.28	0.16
Myogenesis	-0.49	-1.33	0.16
Xenobiotic Metabolism	-0.47	-1.30	0.16
Oestrogen response (early)	-0.47	-1.29	0.16
Hypoxia	-0.46	-1.29	0.16
Hedgehog signalling	-0.65	-1.49	0.16
Complement	-0.46	-1.27	0.16
Bile acid metabolism	0.49	1.33	0.17
IL2 STAT5 signalling	-0.44	-1.24	0.17
IL2 STAT5 signalling	0.43	1.25	0.17
KRAS signalling (up)	-0.45	-1.25	0.18

Postprandial responses at 6h			
**Geneset**	**ES**	**NES**	**p_{adj}**
Acidified milk			
Heme metabolism	-0.46	-1.41	0.01*
Probiotic yoghurt			
Epithelial mesenchymal transition	-0.56	-1.57	0.03*
IL2 STAT5 signalling	-0.48	-1.42	0.04*
Inflammatory response	-0.49	-1.44	0.04*
Glycolysis	-0.48	-1.41	0.04*
IL6 JAK STAT3 signalling	-0.58	-1.57	0.05*
Xenobiotic Metabolism	-0.48	-1.39	0.07*
Allograft rejection	-0.45	-1.33	0.13
Hypoxia	-0.47	-1.36	0.13
Hedgehog signaling	-0.66	-1.58	0.13
Oestrogen response (early)	-0.46	-1.34	0.13
Oestrogen response (late)	-0.47	-1.34	0.13
KRAS signalling (up)	-0.45	-1.32	0.14
Angiogenesis	-0.65	-1.52	0.16
Reactive oxygen species pathway	-0.56	-1.44	0.19
